# Artificial intelligence–enhanced management system for secondary prevention of coronary heart disease: a randomized clinical trial

**DOI:** 10.1186/s12916-026-05135-w

**Published:** 2026-08-18

**Authors:** Zhengqing Ba, Jing Yuan, Sheng Zhao, Mengyuan Liu, Guangzhi Chen, Xiaodan Lian, Fei Yu, Yajing Su, Zuoxiang Wang, Lanshu Yang, Ximei Wang, Yang Wang, Xue Zhang, Wei Zhao, Xiaojin Gao, Yongjian Wu

**Affiliations:** 1 Department of Cardiology, State Key Laboratory of Cardiovascular Disease, National Center for Cardiovascular Diseases, Fuwai Hospital, Chinese Academy of Medical Sciences and Peking Union Medical College, No. 167 Beilishi Road, Xicheng District, Beijing, Beijing, 100037 China; 2 Information Centre, Fuwai Hospital, Chinese Academy of Medical Sciences, Peking Union Medical College, No. 167 Beilishi Road, Xicheng District, Beijing, Beijing, China; 3 Department of Medical Research and Biometrics Center, State Key Laboratory of Cardiovascular Disease, Fuwai Hospital, National Center for Cardiovascular Diseases, Chinese Academy of Medical Sciences and Peking Union Medical College, Beijing, China

**Keywords:** Coronary Artery Disease, Secondary Prevention, Artificial Intelligence, Digital Health

## Abstract

**Background:**

Secondary prevention of coronary heart disease (CHD) remains suboptimal due to fragmented care and therapeutic inertia. While digital health interventions offer a promising strategy, few existing tools provide comprehensive, closed-loop management of multidimensional risk factors without increasing clinician workload. This study aimed to evaluate the efficacy of an artificial intelligence (AI)–enhanced management system (AIM-CHD) in improving multidimensional risk factor control among patients with CHD.

**Methods:**

This single-center, open-label, parallel-group, randomized clinical trial was conducted from November 2024 to June 2025 in China. A total of 1100 adults with confirmed CHD were randomized 1:1 to receive either the AIM-CHD intervention (*n* = 549) or usual care (*n* = 551) for 3 months. The AIM-CHD system featured automated multi-source data capture, guideline-directed risk stratification, and closed-loop feedback via smartphone. The primary outcome was the low-density lipoprotein cholesterol (LDL-C) level at 3 months. Secondary outcomes included goal attainment rates for LDL-C (< 70 mg/dL) and blood pressure (BP, < 130/80 mm Hg), glycated hemoglobin, smoking cessation, body mass index (BMI), medication adherence, and a composite cardiovascular endpoint at 3 months. Analysis was performed on an intention-to-treat (ITT) basis.

**Results:**

Of 1100 randomized participants (mean age 61 years; 75.3% male), 943 (85.7%) completed the 3-month follow-up. The intervention group achieved a significantly lower mean LDL-C level compared with the control group (60.4 ± 23.4 vs. 63.7 ± 26.0 mg/dL), with an adjusted mean difference of − 3.2 mg/dL (95% CI, − 6.2 to − 0.3; *p* = 0.03). Furthermore, participants in the intervention group were more likely to achieve the LDL-C target (71% vs. 64%; RR, 1.10; 95% CI, 1.01–1.20; *p* = 0.03) and the BP target (45% vs. 35%; RR, 1.27; 95% CI, 1.09–1.48; *p* = 0.002). No significant differences were observed for HbA1c, BMI, or medication adherence.

**Conclusions:**

The AIM-CHD system significantly improved short-term lipid and BP control compared with usual care. These findings support interoperable, low-burden digital management systems as a scalable strategy in routine secondary prevention.

**Trial registration:**

ClinicalTrials.gov Identifier: NCT06686056. Registered on 11 November 2024.

**Supplementary Information:**

The online version contains supplementary material available at 10.1186/s12916-026-05135-w.

## Background

Coronary heart disease (CHD) remains a leading cause of global morbidity and mortality [1]. Although effective secondary prevention, through rigorous control of lipids, blood pressure (BP), glucose, and lifestyle, is well established, its real-world implementation remains suboptimal [2–5]. This gap stems from fragmented care continuity, complex multi-risk-factor management, and often low patient engagement, leading to therapeutic inertia and delayed intervention [6, 7].

Digital health technologies, such as smartphone applications and telemedicine, have shown potential in improving medication adherence and supporting lifestyle changes [8–12]. However, existing systematic reviews and randomized trials indicate inconsistent effects on lipid and blood pressure control, and no clear reduction in major adverse cardiovascular events [13–19]. Most current interventions operate in isolation, focus on single risk factors, rely heavily on clinician input, and lack integration across care settings, limiting their scalability and clinical impact [20–22].

**Fig. 1:**
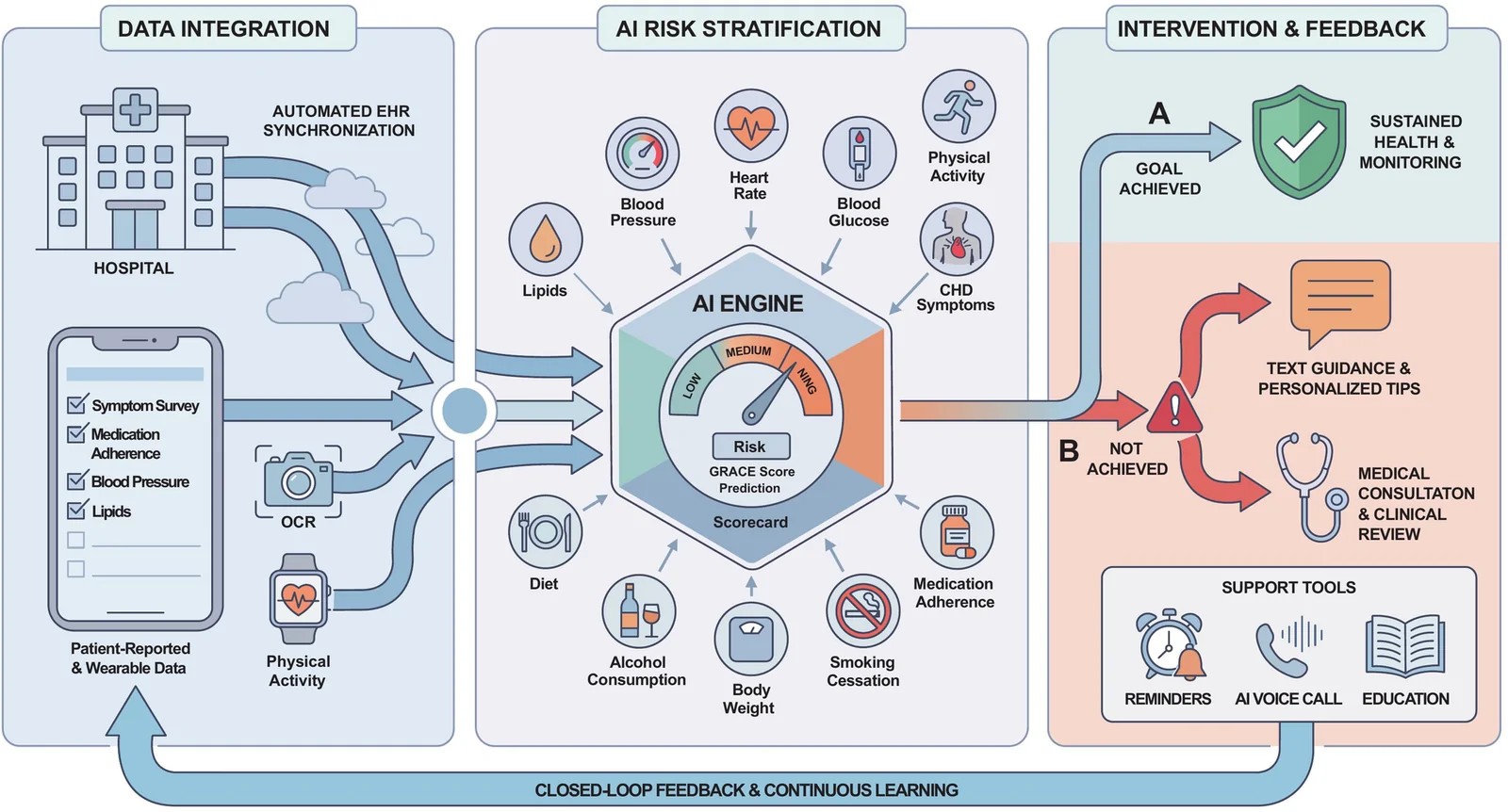
Conceptual framework and operational workflow of the AIM-CHD system. The system integrates three core modules to deliver automated secondary prevention. The Data Integration module synchronizes hospital electronic health records (EHRs) and aggregates multi-source patient data, including OCR-processed reports and wearable metrics. The AI Risk Stratification engine performs dynamic, guideline-based assessments across multidimensional clinical domains. The Intervention & Feedback module executes a closed-loop decision logic. Target attainment maintains monitoring (Path A), while non-attainment (Path B) triggers graded interventions, comprising personalized guidance, safety alerts, and medical referral recommendations, supported by AI tools to ensure a continuous care loop

To address these limitations, we developed the AI-enhanced Management System for Coronary Heart Disease (AIM-CHD), an integrated digital platform designed to automate data capture, enable guideline-directed closed-loop management, and support comprehensive multi-risk-factor control (Fig. 1). Further system specifications are available in the study protocol [23] and in Additional file 1: Fig. S1–S7 and Table S1. This study aimed to evaluate, in a randomized controlled trial, whether the AIM-CHD system improves risk factor control and patient outcomes in CHD secondary prevention.

## Methods

### Study design and participants

This single-center, open-label, parallel-group randomized clinical trial was conducted from November 23, 2024, to June 30, 2025, at the Coronary Heart Disease Center of Fuwai Hospital, National Center for Cardiovascular Diseases, in Beijing, China. The full trial protocol is provided in Additional file 1 and has been published previously [23]. The study was approved by the Ethics Committee of Fuwai Hospital (No. 2024–2422). The trial is reported in accordance with the Consolidated Standards of Reporting Trials (CONSORT) 2025 statement, and the completed checklist is provided in Additional file 1.

Eligible participants were consecutive inpatients aged 18 to 85 years with clinically confirmed CHD who were scheduled for discharge. Inclusion criteria required proficiency in smartphone use (by the patient or a caregiver) and the ability to provide informed consent. Exclusion criteria included severe cognitive impairment, active malignancy or life expectancy of less than 3 months, and multisystem organ failure. Written informed consent was obtained from all participants. Sex and ethnicity were self-reported.

### Randomization

Participants were randomized in a 1:1 ratio to the AIM-CHD group or the usual care group using a central, web-based randomization system. The allocation sequence was generated by an independent statistician using permuted blocks of 4, stratified by baseline low-density lipoprotein cholesterol (LDL-C) levels (< 70 mg/dL or ≥ 70 mg/dL). Due to the behavioural nature of the intervention, participants and care providers were not blinded. However, outcome assessors and data analysts remained masked to group assignment throughout the trial by using de-identified datasets.

### Intervention (AIM-CHD system)

Participants in the intervention group received usual post-discharge care plus access to the AIM-CHD system installed on their personal smartphones. The AIM-CHD system was designed to provide automated, closed-loop secondary prevention management through three key technical components:

1. **Automated Multi-source Data Capture**: The system automatically integrated inpatient data from the hospital information system. For outpatient follow-up, it utilized optical character recognition (OCR) and natural language processing (NLP) to extract values from photos of paper-based laboratory reports and synchronized data from wearable devices via standard application programming interfaces.
2. **Guideline-Directed Risk Stratification**: The system performed dynamic risk assessment for 11 domains (including lipids, BP, glycemia, and lifestyle). Based on these assessments, it tailored the frequency of follow-up and delivered personalized feedback.
3. **Closed-Loop Management**: When treatment targets were not met (e.g., BP ≥ 130/80 mm Hg), the system triggered alerts, educational micro-lessons, and suggestions for medical consultation. Adherence was supported by multimodal reminders, including in-app notifications and intelligent voice calls.

A brief onboarding session was conducted before discharge to verify user proficiency. Technical support was available throughout the study duration. AIM-CHD underwent multiple rounds of testing and iteration before the trial, and a locked, validated version was deployed uniformly to all intervention participants; the algorithm was not modified during the trial to preserve internal validity. All decision support was rule-based and guideline-derived rather than autonomous: the system combined validated GRACE-based risk stratification, recalculated as new data arrived, with automated assessment of 11 clinical and lifestyle domains against predefined thresholds (Additional file 1: Table S1). On-target values prompted continued monitoring, whereas off-target values automatically triggered graded responses (quantitative feedback, recheck prompts, targeted education, medication and adherence reminders, or a recommendation to seek consultation) and safety alerts, without real-time clinician input. Clinicians were involved only when a patient, prompted by the system, attended an outpatient or telemedicine consultation, at which point all therapeutic decisions remained at the treating physician’s discretion; the system never adjusted medication independently. The complete decision logic, thresholds, and interfaces are detailed in the published study protocol [23] and in Additional file 1: Fig. S1–S7 and Table S1.

### Control group

Participants in the control group received usual care, which included standard discharge instructions and routine outpatient follow-up recommendations. To ensure comparable data ascertainment without providing intervention benefits, control participants used a restricted version of the app. This interface allowed for the submission of follow-up outcomes (via questionnaires or photo uploads) but disabled all active intervention features, including risk stratification, feedback, education, and reminders. No blood-pressure or other measurement device was provided to either group; blood-pressure values were obtained without restriction on source (clinic measurement, home self-measurement, or patients’ own wearable devices) and were ascertained identically in both arms.

### Outcomes

The primary outcome was the mean plasma LDL-C level (mg/dL) measured at 3 months (± 14 days) after discharge. If multiple measurements were available within this window, the most recent value was used.

Secondary outcomes assessed at 3 months included: (1) the proportion of participants achieving LDL-C levels less than 70 mg/dL; (2) the proportion achieving target BP, defined as resting blood pressure < 130/80 mm Hg; (3) mean glycated hemoglobin (HbA1c) level; (4) mean body mass index (BMI); (5) smoking cessation (defined as self-reported abstinence for the preceding 30 days); and (6) medication adherence, assessed by patient self-report of the number of days on which prescribed antiplatelet and statin therapy was taken, defined as taking the prescribed therapy on ≥ 80% of days for both drug classes. A composite cardiovascular end point (all-cause death, nonfatal myocardial infarction, nonfatal stroke, or cardiovascular rehospitalization) was also assessed.

Usability was evaluated using the 10-item System Usability Scale (SUS; range, 0-100), and engagement was measured by the completion rate of in-app questionnaires. Safety was monitored through automated system rules for adverse clinical scenarios (e.g., hypotension) and periodic manual review.

### Sample size determination

The sample size was determined based on the primary outcome of plasma LDL-C levels, as detailed in the published protocol [23]. A sample of 950 participants was required to detect a between-group difference of 7.7 mg/dL (SD, 36.7), assuming 90% power and a 2-sided α level of 0.05. To account for a potential 10% attrition rate, the target enrollment was set at 1100 participants.

### Statistical analysis

Primary analyses were performed on an intention-to-treat (ITT) basis, including all randomized participants in their assigned groups. The safety analysis set included all participants who underwent randomization.

For continuous outcomes (e.g., LDL-C, HbA1c), between-group differences were assessed using analysis of covariance (ANCOVA) models, with the 3-month value as the dependent variable and the baseline value as covariates. Results are reported as adjusted mean differences (AMDs) with 95% CIs. Binary outcomes (e.g., control rates) were analyzed using modified Poisson regression with robust error variances to estimate risk ratios (RRs) and 95% CIs. Time-to-event data for the composite cardiovascular outcome were analyzed using Cox proportional hazards regression models adjusted for baseline risk stratification; hazard ratios (HRs) and 95% CIs were calculated. Kaplan-Meier curves were plotted to visualize cumulative event rates.

Missing data for the primary outcome were handled using multiple imputation by chained equations, generating 20 imputed datasets. Sensitivity analyses were conducted to assess the robustness of the findings, including a complete-case analysis and a per-protocol analysis. The per-protocol population was defined as intervention participants who completed at least 1 lipid questionnaire during the intervention period and all control participants with available primary outcome data. Supplementary analyses to assess deviations from the missing-at-random assumption included delta -tipping-point analysis, inverse probability of censoring weighting, and best-case/worst-case scenario analyses.

Prespecified subgroup analyses were conducted to explore potential effect modification by baseline demographic and clinical characteristics (sex, age, education, ethnicity, smoking status, risk stratification, and LDL-C target attainment). Tests for interaction were performed by including a treatment × subgroup interaction term in the models.

All statistical tests were 2-sided, with α = 0.05 indicating statistical significance. Analyses were performed using R, version 4.4.1 (R Foundation for Statistical Computing).

## Results

### Study subjects

**Fig. 2:**
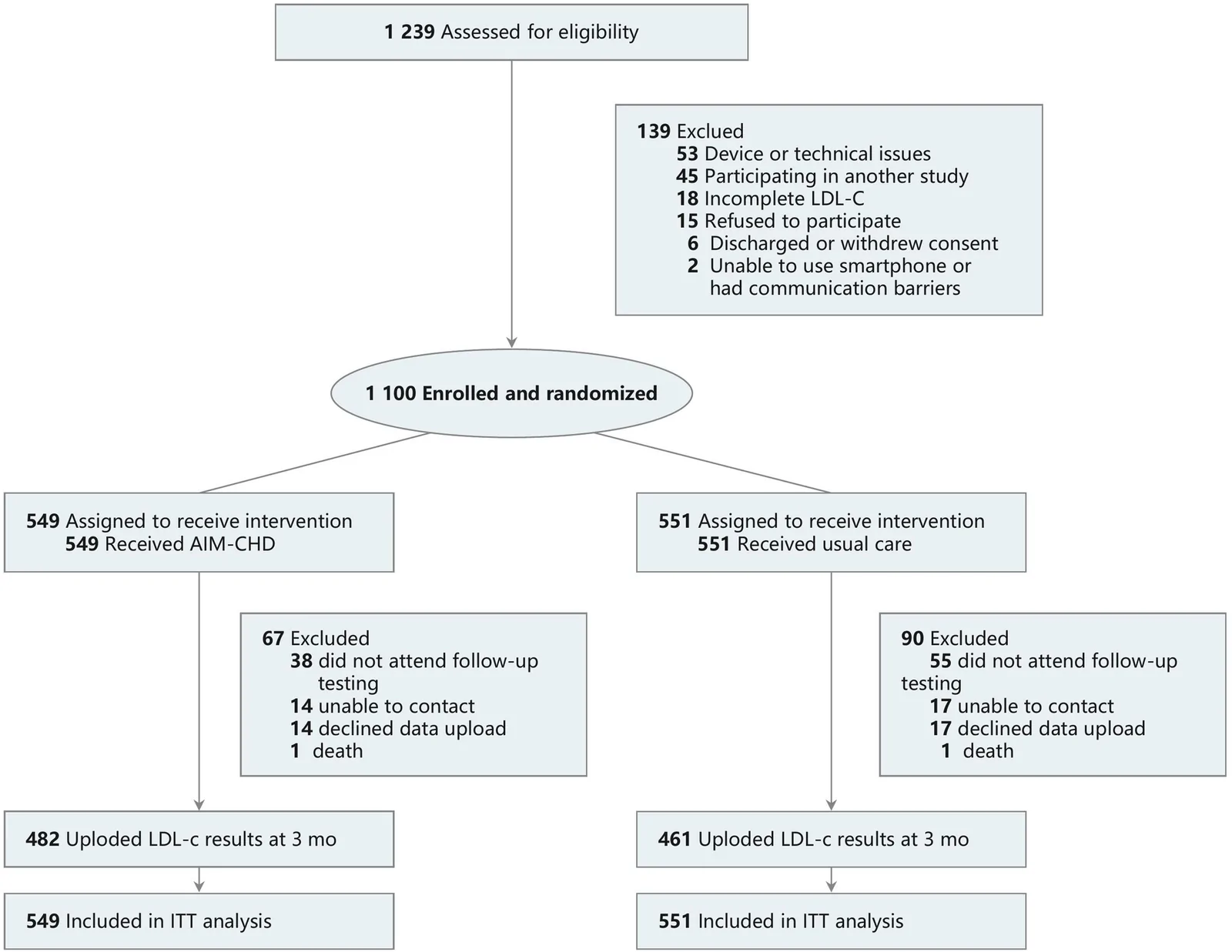
Participant flow diagram. “Device or technical issues” denotes operating-system incompatibility (e.g., early HarmonyOS versions) or non-smartphone devices unable to run the required application. LDL-C indicates low-density lipoprotein cholesterol; AIM-CHD, artificial intelligence–enhanced management system; ITT, intention-to-treat

Between November 23, 2024, and February 18, 2025, 1239 participants were assessed for eligibility. Of these, 139 were excluded, and 1100 (89%) provided informed consent and were randomized to the intervention group (*n* = 549) or the control group (*n* = 551) (Fig. 2). The final follow-up assessment was completed on June 30, 2025.

Baseline characteristics were generally well balanced between groups (Table 1). Participants were recruited from 119 cities across 23 provinces in China (Additional file 1: Table S2). The median (IQR) age was 61 (53–68) years. Among the 1100 participants, 828 (75%) were male and 272 (25%) were female. The majority of participants were Han Chinese (1020 [93%]), with 80 (7%) identifying as non-Han Chinese ethnicities. The mean (SD) baseline low-density lipoprotein cholesterol (LDL-C) level was 78.5 (34.4) mg/dL. Overall, 943 (85.7%) completed the 3-month visit (482 intervention; 461 control). All 1100 randomised participants were included in the intention-to-treat analysis of the primary endpoint (Table 1).

**Table 1:** Baseline Characteristics of Study Participants

Characteristic	Total (*n* = 1100)	Control (*n* = 551)	Intervention (*n* = 549)
**Age**,** years**	61 (53–68)	60 (53–67)	61 (53–68)
**Sex**			
Female	272 (25%)	127 (23%)	145 (26%)
Male	828 (75%)	424 (77%)	404 (74%)
**Ethnicity**			
Han Chinese	1020 (93%)	511 (93%)	509 (93%)
Non-Han Chinese ethnicities	80 (7%)	40 (7%)	40 (7%)
**Education†**			
Low education	421 (38%)	213 (39%)	208 (38%)
Intermediate education	469 (43%)	240 (44%)	229 (42%)
Higher education	210 (19%)	98 (18%)	112 (20%)
**Risk stratification**			
Low risk	898 (82%)	458 (83%)	440 (80%)
Intermediate risk	169 (15%)	80 (15%)	89 (16%)
High risk	33 (3%)	13 (2%)	20 (4%)
**CHD presentation**			
STEMI	41 (4%)	15 (3%)	26 (5%)
NSTEMI	75 (7%)	38 (7%)	37 (7%)
Unstable angina	649 (59%)	320 (58%)	329 (60%)
Stable angina	253 (23%)	136 (25%)	117 (21%)
Asymptomatic CHD	82 (8%)	42 (8%)	40 (7%)
**PCI during hospitalization**	805 (73%)	402 (73%)	403 (73%)
**LDL-C**,** mg/dL**	78.5 (34.4)	79.1 (35.1)	77.9 (33.7)
**LDL-C < 70 mg/dL**	481 (44%)	242 (44%)	239 (44%)
**Hypertension**	751 (68%)	384 (70%)	367 (67%)
**Systolic BP**,** mm Hg**	127.4 (14.8)	128.1 (14.9)	126.7 (14.6)
**Diastolic BP**,** mm Hg**	76.7 (10.9)	77.3 (11.1)	76.0 (10.7)
**BP < 130/80 mm Hg**	475 (44%)	231 (43%)	244 (46%)
**Diabetes mellitus**	420 (38%)	222 (40%)	198 (36%)
**HbA1c**, %*	6.6 (1.2)	6.6 (1.2)	6.7 (1.2)
**BMI**,** kg/m²**	26.0 (3.3)	26.2 (3.3)	25.8 (3.3)
**Smoking history**	579 (53%)	304 (55%)	275 (50%)
**Current non-smoking**,** %**	782 (71%)	370 (67%)	412 (75%)
**Statin therapy‡**	1078 (98%)	541 (98%)	537 (98%)
**PCSK9 inhibitor‡**	174 (16%)	88 (16%)	86 (16%)
**Ezetimibe‡**	749 (68%)	375 (68%)	374 (68%)

Data are n (%), median (IQR), mean (SD). CHD = coronary heart disease; STEMI = ST-segment elevation myocardial infarction; NSTEMI = non–ST-segment elevation myocardial infarction; LDL-C = low-density lipoprotein cholesterol; BP = blood pressure; HbA1c = glycated hemoglobin; BMI = body mass index; PCSK9 = proprotein convertase subtilisin/kexin type 9. *Data not available for all randomised patients, HbA1c, *n* = 988. † Education levels were defined as low (≤ junior middle school), intermediate (high school, technical secondary, or junior college), and high (≥ bachelor’s degree). ‡Medications denote drugs prescribed at hospital discharge from the index admission

### Primary outcome

**Fig. 3:**
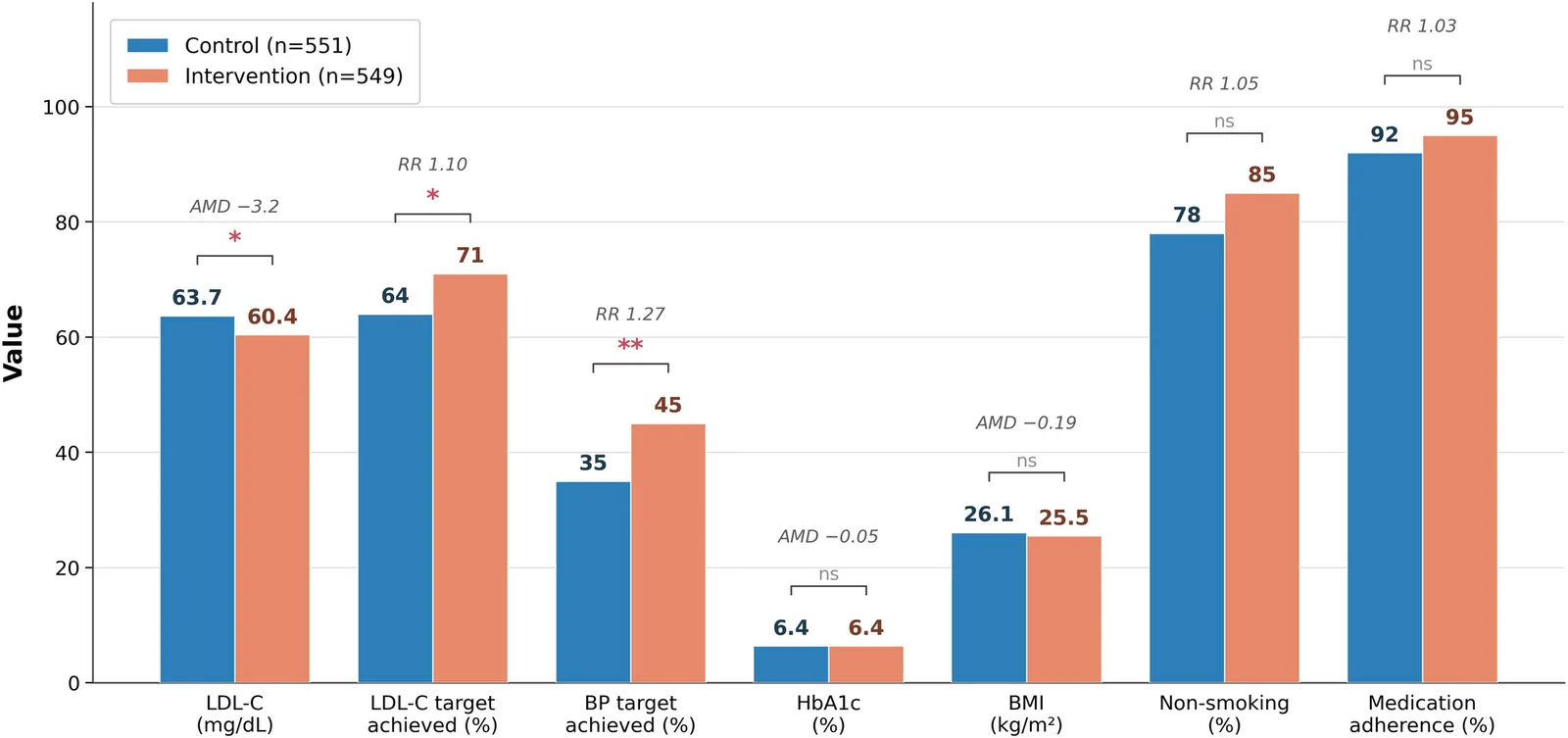
Comparison of outcomes at 3 months between the intervention and control groups in the intention-to-treat population. Data are presented as mean values for continuous outcomes (LDL-C, HbA1c, BMI) and proportions (%) for binary outcomes. Between-group differences were assessed using adjusted mean differences (AMD) for continuous outcomes and risk ratios (RR) for binary outcomes, with corresponding 95% confidence intervals. Significance brackets indicate statistical comparisons between groups. *p < 0.05; **p < 0.01; ns, not significant

At 3 months, the primary outcome of plasma LDL-C level was significantly lower in the intervention group than in the control group (AMD, − 3.2 mg/dL; 95% CI, -6.2 to -0.3 mg/dL; *p* = 0.03) (Fig. 3; Table 2). Results were consistent in the complete-case analysis and showed a larger effect size in the per-protocol analysis (mean difference, − 5.3 mg/dL; 95% CI, − 8.8 to − 1.8 mg/dL) (Table 3; Additional file 1: Table S3).

**Table 2:** Outcomes in the intention-to-treat population with multiple imputations

	*n*	Baseline	Month 3	Estimate (95% CI)	*p* value
**Primary outcome**					
**LDL-C (mg/dL)**					
Control	551	79.1 (35.1)	63.7 (26.0)		
Intervention	549	77.9 (33.7)	60.4 (23.4)	AMD − 3.2 (-6.2 to -0.3)	0.03
**Secondary outcomes**					
**LDL-C target achieved**					
Control	551	242/551 (44%)	352/551 (64%)		
Intervention	549	239/549 (44%)	388/549 (71%)	RR 1.10 (1.01 to 1.20)	0.03
**BP target achieved**					
Control	551	238/551 (43%)	190/551 (35%)		
Intervention	549	253/549 (46%)	245/549 (45%)	RR 1.27 (1.09 to 1.48)	0.002
**Systolic BP (mm Hg)**					
Control	551	128.1 (14.9)	124.8 (13.3)		
Intervention	549	126.7 (14.6)	122.5 (12.3)	AMD − 2.04 (-3.61 to -0.46)	0.01
**Diastolic BP (mm Hg)**					
Control	551	77.3 (11.1)	79.0 (10.3)		
Intervention	549	76.0 (10.7)	76.9 (9.3)	AMD − 1.89 (-3.08 to -0.70)	0.002
**HbA1c (%)**					
Control	551	6.7 (1.2)	6.4 (1.0)	—	—
Intervention	549	6.7 (1.2)	6.4 (1.0)	AMD − 0.05 (− 0.17 to 0.08)	0.46
**BMI (kg/m²)**					
Control	551	26.2 (3.3)	26.1 (3.3)		
Intervention	549	25.8 (3.3)	25.5 (3.4)	AMD − 0.19 (-0.39 to 0.01)	0.06
**Non-smoking**					
Control	551	370/551 (67%)	427/551 (78%)		
Intervention	549	412/549 (75%)	466/549 (85%)	RR 1.05 (1.00 to 1.11)	0.07
**Medication adherence**					
Control	551	—	506/551 (92%)		
Intervention	549	—	520/549 (95%)	RR 1.03 (0.99 to 1.07)	0.12
**Composite Cardiovascular Endpoint**					
Control	528	—	12/528 (2%)		
Intervention	525	—	14/525 (3%)	HR 1.17 (0.54 to 2.54)	0.69

Data are n (%), median (IQR), mean (SD), or n/N (%). LDL-C, low-density lipoprotein cholesterol; BP, blood pressure; SBP, systolic blood pressure; DBP, diastolic blood pressure; HbA1c, glycated hemoglobin; BMI, body-mass index; AMD, adjusted mean difference; RR, risk ratio; HR, hazard ratio

**Table 3:** Outcomes in the per-protocol population

	*n*	Baseline	Month 3	Estimate (95% CI)	*p* value
**Primary outcome**					
**LDL-C (mg/dL)**					
Control	461	77.0 (33.7)	63.7 (26.0)		
Intervention	243	75.1 (31.1)	57.9 (20.2)	AMD − 5.3 (− 8.8 to − 1.8)	0.003
**Secondary outcomes**					
**LDL-C target achieved**					
Control	461	215/461 (47%)	300/461 (65%)		
Intervention	243	107/243 (44%)	183/243 (75%)	RR 1.15 (1.04 to 1.26)	0.005
**BP target achieved**					
Control	474	199/474 (42%)	166/474 (35%)		
Intervention	239	104/239 (44%)	122/239 (51%)	RR 1.47 (1.24 to 1.75)	< 0.001
**Systolic BP (mm Hg)**					
Control	474	128.1 (15.1)	124.5 (13.2)		
Intervention	239	126.1 (13.2)	120.8 (12.8)	AMD − 3.43 (− 5.44 to − 1.42)	< 0.001
**Diastolic BP (mm Hg)**					
Control	474	77.5 (11.3)	78.8 (10.2)		
Intervention	239	76.0 (10.2)	75.6 (8.2)	AMD − 3.23 (− 4.73 to − 1.74)	< 0.001
**HbA1c (%)**					
Control	204	6.9 (1.2)	6.5 (1.0)		
Intervention	122	6.6 (1.2)	6.34(1.0)	AMD − 0.12 (− 0.28 to 0.05)	0.18
**BMI (kg/m²)**					
Control	455	26.2 (3.3)	26.0 (3.3)		
Intervention	236	25.8 (3.0)	25.5 (3.1)	AMD − 0.10 (− 0.34 to 0.14)	0.43
**Non-smoking**					
Control	473	312/473 (66%)	367/473 (78%)		
Intervention	240	180/240 (75%)	212/240 (88%)	RR 1.09 (1.03 to 1.16)	0.004
**Medication adherence**					
Control	468	—	434/468 (93%)		
Intervention	238	—	228/238 (96%)	RR 1.03 (1.00 to 1.07)	0.08
**Composite Cardiovascular Endpoint**					
Control	528	—	12/528 (2%)		
Intervention	237	—	2/237 (1%)	HR 0.37 (0.08 to 1.65)	0.21

Data are n (%), median (IQR), mean (SD), or n/N (%). LDL-C, low-density lipoprotein cholesterol; BP, blood pressure; SBP, systolic blood pressure; DBP, diastolic blood pressure; HbA1c, glycated hemoglobin; BMI, body-mass index; AMD, adjusted mean difference; RR, risk ratio; HR, hazard ratio

### Secondary outcomes

Compared with the control group, the intervention group showed significantly higher rates of LDL-C target (71% vs. 64%; RR, 1.10 [95% CI, 1.01–1.20]; *p* = 0.03) and the BP target (45% vs. 35%; RR, 1.27 [95% CI, 1.09–1.48]; *p* = 0.002). No significant between-group differences were observed for glycated hemoglobin (HbA1c) levels, BMI, current nonsmoking status, medication adherence, or the composite cardiovascular end point (Table 2). In per-protocol analyses, the rate of self-reported smoking abstinence was significantly higher in the intervention group (Table 3).

### Subgroup and sensitivity analyses

Prespecified subgroup analyses for the primary and key secondary end points showed no evidence of effect modification by sex, age, education, ethnicity, smoking status, baseline risk stratification, or baseline LDL-C target attainment (all p for interaction > 0.05) (Additional file 1: Fig. S8–S14). Participants with missing 3-month LDL-C data differed from those with complete data in educational attainment, baseline clinical profile, and follow-up engagement (Additional file 1: Tables S4 and S5). However, sensitivity analyses addressing missing data, including delta tipping-point analyses, inverse probability of censoring weighting, and common-bounds imputations, yielded results consistent with the primary intention-to-treat analysis (Additional file 1: Tables S6–S8). Missingness for all endpoints, overall and by group, is summarized in Additional file 1: Table S9.

### Process measures, engagement, and safety

During the management period (days 1–76), the rate of outpatient follow-up was significantly higher in the intervention group than in the control group (86.4% vs. 72.6%; risk ratio, 1.19; 95% CI, 1.12–1.27; *p* < 0.001). Across follow-up, 24,601 outcome entries were captured, 75.4% of which were automated (Additional file 1: Table S10). Of 5498 AI voice call attempts, 2720 (49.5%) were successfully connected. Usability scores were favorable, with a mean (SD) System Usability Scale score of 75.0 (15.9). In an exploratory analysis of attribution, outpatient revisits combined with questionnaire completion were associated with the greatest reduction in LDL-C levels (mean difference, − 9.1 mg/dL; 95% CI, − 14.2 to − 4.0 mg/dL), whereas revisits without questionnaire completion were associated with a smaller but significant improvement (− 6.7 mg/dL; 95% CI, − 10.7 to − 2.7 mg/dL) (Additional file 1: Table S11).

Adverse clinical events were uncommon and similar between groups (14 [2.6%] in the intervention group vs. 12 [2.2%] in the control group). Two deaths occurred (1 in each group). No adverse events were deemed related to the study intervention (Additional file 1: Table S12).

## Discussion

In this randomized clinical trial of a digital therapeutic system for secondary prevention of CHD, the AIM-CHD intervention significantly reduced LDL-C levels and blood pressure over a 3-month period compared with usual care. Although trends toward improvement were observed for smoking cessation, glycemic control, BMI, and medication adherence, these did not reach statistical significance. These findings suggest that a fully automated, closed-loop, multimodal digital intervention may help deliver scalable, coordinated risk factor management.

The reduction in LDL-C levels observed in this study (AMD, − 3.2 mg/dL) is small in absolute terms and should be interpreted in context. Both groups were discharged on at least moderate-intensity statin therapy, with a proportion also receiving PCSK9 inhibitors, and baseline LDL-C was already relatively well controlled (mean, 78.5 mg/dL), so achieving a statistically significant further reduction against this near-ceiling background is non-trivial. This finding aligns with results from the TEXT ME trial, which reported a reduction of approximately 5.0 mg/dL with semi-personalized SMS over 6 months [13].

However, our study extends the evidence base by demonstrating efficacy using a fully automated app-based model rather than text messaging. Prior app-based interventions have largely focused on lifestyle modification or medication adherence, with inconsistent effects on physiological biomarkers [11, 22, 24–26]. By targeting objective biometric endpoints (LDL-C and BP) through direct EHR integration and automated feedback, AIM-CHD addresses a critical gap identified in previous trials, such as the TEXTMEDS study [16], which found that generic broadcast messaging offered diminishing returns in information-saturated environments.

The divergence in outcomes between successful and null digital trials suggests that the primary driver of biomarker improvement is not the communication channel itself, but the underlying structure that transforms patient data into protocolized clinical actions [11, 17, 18, 20, 25, 27]. Pharmacist-led models have achieved substantial LDL-C reductions through systematic titration [28], yet these are resource-intensive. AIM-CHD was designed to automate this principle by converting measurements into action via logic-based prompts for rechecks and feedback. Our exploratory analysis supports this mechanism: participants who engaged with the feedback loop by submitting lipid results (mean difference, − 9.1 mg/dL) or attending clinic visits (mean difference, − 6.7 mg/dL) derived substantially greater benefit. Notably, this benefit emerged despite near-ceiling medication adherence in both arms (95% vs. 92%), and was accompanied by a higher outpatient revisit rate (86.4% vs. 72.6%; *p* < 0.001) and reinforced lifestyle modification. The attenuation of the overall effect size likely reflects partial activation of this loop (only 44.3% uploaded lipid results) and the high baseline control rate. The larger effect observed in the per-protocol analysis and among patients with higher baseline LDL-C supports the potential yield of broader engagement and targeted application.

Heterogeneity was evident across secondary outcomes. Significant improvements were seen in BP control, a notable finding given that prior SMS-only trials often reported null effects on systolic BP [17, 18]. This may be related to the platform’s explicit goal feedback and wearable synchronization. Conversely, the neutral results for HbA1c and weight likely reflect the longer time course required for these outcomes to change rather than a lack of efficacy. HbA1c reflects average glycaemic exposure over the preceding 8–12 weeks and is relatively insensitive to short-term change [29], whereas meaningful weight loss generally requires sustained lifestyle modification over ≥ 6 months [30]; neither outcome was targeted by a specific titration protocol in the current algorithm, and the ongoing 12-month follow-up will allow them to be reassessed.

Although a formal cost-effectiveness analysis was not prespecified for this efficacy trial and is planned alongside the longer-term follow-up, we outline the contrasting cost structures of the two approaches. AIM-CHD entails a one-time development cost (approximately ¥1.0 million [≈ US$140 000]) that is highly amortizable. The same platform has already been deployed across more than 20 hospitals through a separate study, so the per-patient share of this fixed cost falls steeply with scale. Beyond development, the marginal cost per patient approaches zero: onboarding requires roughly 10 min of existing nursing time before discharge, while subsequent reminders, recheck prompts, risk stratification, feedback, and education are fully automated; ongoing operation requires only a small fixed team for maintenance and user support, a cost that does not grow with patient volume. By contrast, usual care relies on clinician- and nurse-delivered follow-up calls and manual interpretation of laboratory reports. The labor scales linearly with patient volume and is vulnerable to fatigue, inconsistency, and error, without real-time response. The system’s high usability scores (mean SUS, 75.0) and 75.4% automated data capture further support its feasibility for routine, scalable implementation.

This study has several limitations. First, although participants were recruited from 119 cities across 23 provinces, the single-center design and the predominantly Han Chinese cohort (93%) may limit the generalizability of these findings to other ethnic populations and health-care systems; external validation in multicenter, multiethnic settings is therefore warranted. Second, the open-label design may introduce performance bias. However, this risk is mitigated by the use of objective biomarker endpoints (LDL-C and BP) and by masking of outcome assessors and analysts. Third, the 3-month follow-up period is short and was insufficient to assess the durability of effects or changes in hard clinical end points; a 12-month follow-up is prospectively planned and ongoing, and will be reported separately. Fourth, the incomplete activation of the automated feedback loop represents a key implementation challenge that future iterations must address. Fifth, 14.3% of participants had missing primary-outcome data; however, comprehensive sensitivity analyses supported the robustness of the findings.

## Conclusions

In conclusion, in this randomized clinical trial, the AIM-CHD system demonstrated that a fully automated, closed-loop digital health platform can yield simultaneous improvements in lipid and blood pressure control among patients with CHD. This approach may represent a pragmatic and scalable model for post-discharge care. While the primary endpoint provides confirmatory evidence for short-term risk-factor control, findings regarding long-term durability and hard clinical endpoints remain hypothesis-generating and warrant confirmation in longer-term, multicenter studies.

## Supplementary Information

Below is the link to the electronic supplementary material.

Supplementary Material 1: Additional file 1: Supplementary tables and figures, study protocol and CONSORT 2025 checklist. Table S1 – Management targets and safety-alert criteria of the AIM-CHD system. Table S2 – Provincial distribution of the study cohort. Table S3 – Outcomes in the intention-to-treat population, complete cases. Tables S4–S5 – Characteristics of participants with and without 3-month LDL-C data, overall and by randomised group. Tables S6–S8 – Sensitivity analyses for missing 3-month LDL-C data (Δ-tipping-point analysis, inverse probability of censoring weighting, and bounds analysis). Table S9 – Missing data by endpoint and randomised group. Table S10 – Overview of data collection across all endpoints during follow-up. Table S11 – Effect on LDL-C by joint state of outpatient revisit, questionnaire completion and medication indicators. Table S12 – Clinical events within 3 months. Figs. S1–S7 – Operational logic, management indicators and user interfaces of the AIM-CHD system (software backend, main interface, questionnaire, health-indicator recording, medication reminders and health-education section). Figs. S8–S14 – Subgroup analyses at 3 months for LDL-C, lipid target attainment, blood-pressure target attainment, non-smoking status, HbA1c, BMI and medication adherence. Study protocol – AIM-CHD trial protocol, version 1.2, 22 October 2024. CONSORT 2025 checklist.

## Data Availability

The datasets used and/or analysed during the current study are available from the corresponding author on reasonable request.

## Abbreviations

AI: Artificial intelligence
AIM-CHD: Artificial intelligence–enhanced management system for coronary heart disease
AMD: Adjusted mean difference
ANCOVA: Analysis of covariance
BMI: Body mass index
BP: Blood pressure
CHD: Coronary heart disease
CI: Confidence interval
CONSORT: Consolidated Standards of Reporting Trials
EHR: Electronic health record; HbA1c: Glycated haemoglobin
HR: Hazard ratio
IPCW: Inverse probability of censoring weighting
IQR: Interquartile range
ITT: Intention-to-treat
LDL-C: Low-density lipoprotein cholesterol
NLP: Natural language processing
NSTEMI: Non–ST-segment elevation myocardial infarction
OCR: Optical character recognition
PCI: Percutaneous coronary intervention
PCSK9: Proprotein convertase subtilisin/kexin type 9
RR: Risk ratio
SD: Standard deviation
STEMI: ST-segment elevation myocardial infarction
SUS: System Usability Scale
