# Supplementary material for: Artificial intelligence–enhanced management system for secondary prevention of coronary heart disease: a randomized clinical trial
Source: BMC Med. 2026 Aug 18;24:449. doi: 10.1186/s12916-026-05135-w (PMC13488134; doi:10.1186/s12916-026-05135-w)
Supplement: Supplementary file 1 [file 12916_2026_5135_MOESM1_ESM.pdf]

Additional file 1

Part 1. Supplementary tables and figures

eTable 1.Management Targets and Safety alert Criteria in AIM-CHD. 2
eTable 2. Provincial distribution of the study cohort (N=1,100). 4
eTable 3. Outcomes in the intention-to-treat population with complete cases 5
eTable 4. Characteristics of participants with and without 3-month LDL-C data 7
eTable 5. Characteristics of participants without 3-month LDL-C data: intervention vs control 9
eTable 6. Δ-tipping-point sensitivity analysis...11
eTable 7. Inverse probability censoring weighting (IPCW) analysis of 3-month LDL-C ...11
eTable 8. Bounds analysis for missing 3-month LDL-C values 11
eTable 9. Missing data by endpoint and randomized group 13
eTable 10. Overview of data collection across all endpoints during the follow-up period 13
eTable 11. Effect on LDL-C (mg/dL) with joint state of revisit & questionnaire and medication indicators (N = 943) 14
eTable 12. Clinical Events Within 3 Months (Intention-To-Treat Population).................. 15
eFigure 1. Operational logic and management indicators of the AIM-CHD..................... 16
eFigure 2. Software Backend.. 17
eFigure 3. Software Main Interface... 18
eFigure 4. Questionnaire Interface 19
eFigure 5. Health Indicators Recording 20
eFigure 6. Medication Reminders... 21
eFigure 7. Health Education Section 22
eFigure 8. Subgroup analysis of LDL-C at 3-month 23
eFigure 9. Subgroup analysis of lipid target attainment at 3-month 24
eFigure 10. Subgroup analysis of blood-pressure target attainment at 3-month 25
eFigure 11. Subgroup analysis of Non-smoking status at 3-month 26
eFigure 12. Subgroup analysis of HbA1c at 3-month... 27
eFigure 13. Subgroup analysis of BMI at 3-month 28
eFigure 14. Subgroup analysis of medication adherence at 3-month 29

Part 2. Study protocol (AIM-CHD trial protocol, version 1.2, 22 October 2024)

Part 3. CONSORT 2025 checklist

eTable 1.Management Targets and Safety alert Criteria in AIM-CHD.

Domain	Indicator	Target	Threshold for medical adjustment	Safety alert criteria
Lipids	LDL-C	<70 mg/dL	≥70 mg/dL	NA
Blood Pressure	Systolic blood pressure	<130 mmHg	≥130 mmHg	serum creatinine increase >30% from discharge; SBP <90 or >160 mmHg; DBP <60 or >120 mmHg
	Diastolic blood pressure	<80 mmHg	≥80 mmHg	
Heart Rate	Resting heart rate	55 – 60 bpm	≥70 bpm	Heart rate <50 bpm or >100 bpm
Blood Glucose	Hemoglobin A1c	<7.0%	≥7.0%	Fasting blood glucose≤72 or ≥252 mg/dL; symptoms of hypoglycemia
	Fasting blood glucose	80 – 130 mg/dL	≥130 mg/dL	
	2-hour postprandial glucose	<180 mg/dL	≥180 mg/dL	
CHD Symptoms	Symptoms	None	NA	chest pain/tightness, palpitations, or dyspnea within the past month; prolonged, intermittent, or spontaneous episodes
Medication Adherence	Antiplatelet agents	≥80% adherence	NA	Bleeding (e.g., eye, nasal, or oral bleeding, black/red stool); Hb decrease >30 g/L or <100 g/L
	Statins	≥80% adherence	NA	muscle pain/weakness; ALT >120 U/L; CK >1000 U/L
Smoking Cessation	Smoking	Complete cessation	NA	NA
Body Weight	BMI	20–25 kg/m² (18–64 years); 22–26.9 kg/m² (≥65 years)	NA	NA
Alcohol Consumption	Intake	abstain if no prior use; <100 g/week if prior use	NA	NA
Physical Activity	Exercise	30–60 minutes of moderate-intensity, 5 days/week	NA	NA
Diet	Diet	Rich in fruits, vegetables, legumes, fiber, polyunsaturated fats, nuts, fish	NA	NA

LDL-C, Low-Density Lipoprotein Cholesterol; CHD, coronary heart disease; BMI, body mass index. Adapted from Ba Z, Zhao S, Liu M, et al. BMJ Open 2025;15:e105597. Licensed under

CC BY-NC 4.0 (http://creativecommons.org/licenses/by-nc/4.0/).

eTable 2. Provincial distribution of the study cohort (N=1,100).

Province/Region	Frequency, n	Percent, %
Hebei	293	26.6
Beijing	271	24.6
Shandong	141	12.8
Inner Mongolia	83	7.5
Shanxi	68	6.2
Heilongjiang	47	4.3
Henan	34	3.1
Liaoning	34	3.1
Xinjiang	27	2.5
Jilin	21	1.9
Unknown	19	1.7
Anhui	14	1.3
Shaanxi	13	1.2
Tianjin	10	0.9
Jiangsu	6	0.5
Jiangxi	4	0.4
Hunan	4	0.4
Gansu	3	0.3
Guizhou	2	0.2
Qinghai	2	0.2
Yunnan	1	0.1
Guangdong	1	0.1
Zhejiang	1	0.1
Hubei	1	0.1
**Total**	**1100**	**100**

eTable 3. Outcomes in the intention-to-treat population with complete cases

	n	Baseline	3 Months	Adjusted effect (95% CI)	p value
**Primary outcome**					
**LDL-C (mg/dL)**					
Control	461	77.0 (33.7)	63.7 (26.0)		
Intervention	482	77.0(31.9)	60.4 (23.4)	AMD -3.2 (-6.2 to -0.3)	0.03
**Secondary outcomes**					
**LDL-C target achieved**					
Control	461	215/461 (47%)	300/461 (65%)		
Intervention	482	212/482 (44%)	346/482 (72%)	RR 1.10 (1.01–1.20)	0.02
**BP target achieved**					
Control	474	199/474 (42%)	166/474 (35%)		
Intervention	501	221/501 (44%)	226/501 (45%)	RR 1.29 (1.10–1.51)	0.001
**Systolic BP (mm Hg)**					
Control	474	128.1 (15.1)	124.5 (13.2)		
Intervention	501	126.6 (14.5)	122.4 (12.2)	AMD -1.85 (-3.42 to -0.28)	0.02
**Diastolic BP (mm Hg)**					
Control	474	77.5 (11.3)	78.8 (10.2)		
Intervention	501	75.8 (10.7)	76.7 (9.2)	AMD -1.88 (-3.10 to -0.66)	0.003
**HbA1c (%)**					
Control	204	6.9 (1.2)	6.5 (1.0)		
Intervention	206	6.8 (1.4)	6.4 (1.1)	AMD -0.06 (-0.21 to 0.09)	0.44
**BMI (kg/m²)**					
Control	455	26.2 (3.3)	26.0 (3.3)		
Intervention	495	25.8 (3.4)	25.5 (3.4)	AMD -0.17 (-0.37 to 0.03)	0.09
**Non-smoking**					
Control	473	312/473 (66%)	367/473 (78%)		
Intervention	501	375/501 (75%)	429/501 (86%)	RR 1.06 (1.00–1.11)	0.04
**Medication adherence**					
Control	468	—	434/468 (93%)		
Intervention	500	—	475/500 (95%)	RR 1.02 (0.99–1.06)	0.14
**Composite Cardiovascular Endpoint**					
Control	528	—	12/528 (2%)		
Intervention	525	—	14/525 (3%)	HR 1.17 (0.54–2.53)	0.69

Data are n (%), median (IQR), mean (SD), or n/N (%). LDL-C, low-density lipoprotein

cholesterol; BP, blood pressure; SBP, systolic blood pressure; DBP, diastolic blood pressure; HbA1c, glycated hemoglobin; BMI, body-mass index; AMD, adjusted mean difference; RR, risk ratio; HR, hazard ratio.

eTable 4. Characteristics of participants with and without 3-month LDL-C data

Variable	Had LDL-c At 3 Month (n=943)	Missing LDL-c At 3 Month (n=157)	P value
**Randomization group**			**0.050**
Control	461 (48.9%)	90 (57.3%)	
Intervention	482 (51.1%)	67 (42.7%)	
**Age (years)**	60 (53–68)	61 (55–68)	0.382
**Sex**			0.716
Female	235 (24.9%)	37 (23.6%)	
Male	708 (75.1%)	120 (76.4%)	
**Ethnicity**			0.064
Non-Han Chinese ethnicities	63 (6.7%)	17 (10.8%)	
Han Chinese	880 (93.3%)	140 (89.2%)	
**Education**			**0.045**
Intermediate education	403 (42.7%)	66 (42.0%)	
Low education	350 (37.1%)	71 (45.2%)	
Higher education	190 (20.1%)	20 (12.7%)	
**Risk stratification**			0.774
Intermediate risk	142 (15.1%)	27 (17.2%)	
Low risk	773 (82.0%)	125 (79.6%)	
High risk	28 (3.0%)	5 (3.2%)	
**CHD presentation**			**0.001**
NSTEMI	64 (6.8%)	11 (7.0%)	
STEMI	39 (4.1%)	2 (1.3%)	
Unstable angina	556 (59.0%)	93 (59.2%)	
Stable angina	225 (23.9%)	28 (17.8%)	
Asymptomatic CHD	59 (6.3%)	23 (14.6%)	
**PCI during hospitalization**	718 (76.1%)	87 (55.4%)	**<0.001**
**LDL-C (mg/dL)**	76.8 (32.8)	87.6 (42.1)	**<0.001**
**LDL-C at target (%)**	427 (45.3%)	54 (34.4%)	**0.011**
**Statin therapy**	925 (98.1%)	153 (97.5%)	0.825
**PCSK9 inhibitor**	142 (15.1%)	32 (20.4%)	0.091
**Ezetimibe**	653 (69.2%)	96 (61.1%)	**0.044**
**Hypertension**	651 (69.0%)	100 (63.7%)	0.183
**Systolic BP (mmHg)**	127.33 (14.65)	127.78 (15.40)	0.725
**Diastolic BP (mmHg)**	76.50 (10.99)	77.56 (10.29)	0.264
**Baseline BP at target (%)**	413 (44.8%)	62 (40.5%)	0.319
**Diabetes mellitus**	357 (37.9%)	63 (40.1%)	0.588
**HbA1c (%)**	6.61 (1.19)	6.81 (1.38)	0.077

**BMI (kg/m²)**	25.90 (3.25)	26.69 (3.61)	**0.006**
**Smoking history**	485 (51.4%)	94 (59.9%)	**0.050**
**Current non-smoking (%)**	678 (71.9%)	104 (66.2%)	0.148
**Outpatient revisit times***			**<0.001**
0	162 (17.6%)	55 (40.7%)	
1	498 (54.0%)	55 (40.7%)	
≥2	263 (28.5%)	25 (18.5%)	
**Total outpatient revisit visits***	1.29 (1.07)	0.87 (0.97)	**<0.001**
**Questionnaire completion times***			**<0.001**
0	647 (68.6%)	157 (100.0%)	
1	216 (22.9%)	0 (0.0%)	
≥2	80 (8.5%)	0 (0.0%)	
**Wearable device use***	24 (2.5%)	1 (0.6%)	

*Outpatient revisit times, total outpatient revisit times, questionnaire completion times, and wearable device use were counted during the intervention period (days 1–76).

eTable 5. Characteristics of participants without 3-month LDL-C data: intervention vs control

Variable	Control Group (n=90)	Intervention Group (n=67)	P value
**Age (years)**	60 (54–68)	63 (56–69)	0.169
**Sex**			0.645
Female	20 (22.2%)	17 (25.4%)	
Male	70 (77.8%)	50 (74.6%)	
**Ethnicity**			0.154
Non-Han Chinese ethnicities	7 (7.8%)	10 (14.9%)	
Han Chinese	83 (92.2%)	57 (85.1%)	
**Education**			0.912
Intermediate education	37 (41.1%)	29 (43.3%)	
Low education	42 (46.7%)	29 (43.3%)	
Higher education	11 (12.2%)	9 (13.4%)	
**Risk stratification**			0.744
Intermediate risk	15 (16.7%)	12 (17.9%)	
Low risk	73 (81.1%)	52 (77.6%)	
High risk	2 (2.2%)	3 (4.5%)	
**CHD presentation**			0.738
NSTEMI	6 (6.7%)	5 (7.5%)	
STEMI	1 (1.1%)	1 (1.5%)	
Unstable angina	53 (58.9%)	40 (59.7%)	
Stable angina	19 (21.1%)	9 (13.4%)	
Asymptomatic CHD	11 (12.2%)	12 (17.9%)	
**PCI during hospitalization**	49 (54.4%)	38 (56.7%)	0.777
**LDL-C (mg/dL)**	89.6 (40.5)	84.6 (44.4)	0.435
**LDL-C at target (%)**	27 (30.0%)	27 (40.3%)	0.179
**Statin therapy**	87 (96.7%)	66 (98.5%)	0.832
**PCSK9 inhibitor**	17 (18.9%)	15 (22.4%)	0.59
**Ezetimibe**	57 (63.3%)	39 (58.2%)	0.515
**Hypertension**	61 (67.8%)	39 (58.2%)	0.218
**Systolic BP (mmHg)**	128.83 (15.38)	126.36 (15.42)	0.321
**Diastolic BP (mmHg)**	78.07 (9.99)	76.89 (10.70)	0.486
**Baseline BP at target (%)**	35 (40.2%)	27 (40.9%)	0.932
**Diabetes mellitus**	37 (41.1%)	26 (38.8%)	0.771
**HbA1c (%)**	6.77 (1.44)	6.86 (1.29)	0.715
**BMI (kg/m²)**	27.06 (3.40)	26.18 (3.84)	0.131
**Smoking history**	52 (57.8%)	42 (62.7%)	0.535
**Current non-smoking (%)**	57 (63.3%)	47 (70.1%)	0.372

**Outpatient recheck times***			0.214
0	36 (46.2%)	19 (33.3%)	
1	27 (34.6%)	28 (49.1%)	
≥2	15 (19.2%)	10 (17.5%)	
**Total outpatient follow-up visits***	0.86 (1.08)	0.89 (0.82)	0.834
**Questionnaire completion times***			
0	90 (100.0%)	67 (100.0%)	-
1	0 (0.0%)	0 (0.0%)	
≥2	0 (0.0%)	0 (0.0%)	
**Wearable device use***	0 (0.0%)	1 (1.5%)	0.427

***Outpatient revisit times, total outpatient revisit times, questionnaire completion times, and wearable device use were counted during the intervention period (days 1–76).

eTable 6. Δ-tipping-point sensitivity analysis

Scenario	Δ shift (mg/dL) †	β estimate ‡	95% CI	p value
Equal downward shift (Δ_treat = Δ_control = −d)	−8.1	−2.9	−5.9, +0.0	0.051
Equal upward shift (Δ_treat = Δ_control = +d)	≥ +38.6 (tested to +38.61)	−4.8	−8.2, −1.4	0.006
Intervention only upward (Δ_treat = +d, Δ_control = 0)	2.7	−2.9	−5.9, +0.0	0.051
Worst-case (Intervention up, Control down) (Δ_treat = +d, Δ_control = −d)	±1.2	−2.9	−5.9, +0.0	0.051

† Δ shift indicates the additional offset imposed on imputed LDL-C values for participants with missing outcomes.

‡ β estimate = treatment effect estimate (Intervention vs Control).

Note: Threshold rows are reported at the minimal Δ where p ≥ 0.05; due to rounding, these appear as p≈0.051.

eTable 7. Inverse probability censoring weighting (IPCW) analysis of 3-month LDL-C

Variable	β estimate	Robust SE	95% CI	p value
Intervention (vs control)	−3.9	1.5	−7.0, −0.8	0.020
Baseline LDL-C	+10.4	1.5	+8.9, +12.4	<0.001

Probabilities of being observed were estimated from a logistic regression model including groups, age, sex, BMI, smoking, baseline LDL-C, CAD presentation, PCI status, and lipid-lowering therapies.

To address potential bias due to missing 3-month LDL-C values (mg/dL), we estimated the probability of being observed given baseline demographic, clinical, and treatment variables using logistic regression. Stabilized weights (1/p) were applied to complete cases, truncated at 0.01 and 0.99. Weighted ANCOVA with robust (HC3) standard errors showed that the intervention group had significantly lower LDL-C compared with the control group (β = −3.9, 95% CI −7.0 to −0.8, p = 0.020), consistent with the multiple imputation and complete-case analyses.

eTable 8. Bounds analysis for missing 3-month LDL-C values

Scenario	Assumption for missing values (both arms)	β estimate	SE	p value
Common lower bound	All missing values set to 63.7 mg/dL	−2.6	1.3	0.047

Scenario	Assumption for missing values (both arms)	β estimate	SE	p value
Common upper bound	All missing values set to 78.4 mg/dL	−3.2	1.4	0.018

We used a common-bounds strategy because the missing subgroups in the intervention and control arms showed similar characteristics and received no distinct follow-up. The lower bound (63.7 mg/dL) is defined as the intervention arm's observed mean LDL-C at the 3-month follow-up; the upper bound (78.4 mg/dL) is defined as the overall cohort's baseline mean LDL-C (unfavorable). Estimates are from an ANCOVA adjusted for baseline LDL-C; $\beta < 0$ favors the intervention. Two-sided p values are reported without multiplicity adjustment. Under both extreme, arm-symmetric MNAR scenarios, the treatment effect remains statistically significant.

eTable 9. Missing data by endpoint and randomized group

Endpoint	Total missing, n (%)	Control, n (%)	Intervention, n (%)	Difference, % (95% CI)	P value
LDL-C, 3 mo (primary)	157 (14.3)	90 (16.3)	67 (12.2)	4.1 (−0.2 to 8.4)	.061
Lipid target attainment	157 (14.3)	90 (16.3)	67 (12.2)	4.1 (−0.2 to 8.4)	.061
BP target attainment	125 (11.4)	77 (14.0)	48 (8.7)	5.2 (1.3 to 9.2)	.008
Non-smoking at follow-up	126 (11.5)	78 (14.2)	48 (8.7)	5.4 (1.5 to 9.3)	.006
Medication adherence	132 (12.0)	83 (15.1)	49 (8.9)	6.1 (2.1 to 10.1)	.002
HbA1c, 3 mo	112 (10.2)	57 (10.3)	55 (10.0)	0.3 (−3.4 to 4.1)	.937
BMI, 3 mo	150 (13.6)	96 (17.4)	54 (9.8)	7.6 (3.4 to 11.8)	<.001
Rehospitalization event	47 (4.3)	23 (4.2)	24 (4.4)	−0.2 (−2.8 to 2.4)	.990

eTable 10. Overview of data collection across all endpoints during the follow-up period

Dataset	Hospital revisit	Patient-reported	Wearable	Staff-assisted	Total
DBP	727 (12.5%)	3496 (60.0%)	818 (14.0%)	786 (13.5%)	5827
SBP	719 (12.3%)	3501 (60.1%)	818 (14.0%)	786 (13.5%)	5824
LDL-c	587 (25.1%)	805 (34.5%)	0	942 (40.4%)	2334
Weight	171 (7.4%)	1365 (59.2%)	0	769 (33.4%)	2305
Antiplatelet	0	1184 (59.7%)	0	800 (40.3%)	1984
Lipid drugs	0	1153 (59.0%)	0	800 (41.0%)	1953
Events	133 (7.2%)	1665 (89.7%)	0	58 (3.1%)	1856
Smoking	0	911 (53.0%)	0	807 (47.0%)	1718
HbA1c	243 (30.4%)	257 (32.1%)	0	300 (37.5%)	800
Total	2580 (10.5%)	14337 (58.3)	1636(6.7%)	6048(24.6%)	24601

eTable 11. Effect on LDL-C (mg/dL) with joint state of revisit & questionnaire and medication indicators (N = 943)

Predictor	Estimate (β)	Std. Error	t value	p value	95% CI
Intercept	62.6	6.0	10.5	<0.001	[50.9, 74.3]
Intervention arm (vs control)	−1.3	1.8	−0.7	0.47	[−4.9, 2.2]
revisit_no_questionnaire (vs no_revisit)	−6.7	2.0	−3.3	0.001	[−10.7, −2.7]
revisit_questionnaire (vs no_revisit)	−9.1	2.6	−3.5	<0.001	[−14.2, −4.0]
Baseline LDL-C (mg/dL)	0.30	0.02	12.3	<0.001	[0.25, 0.34]
Statin use (yes vs no)	−10.5	5.5	−1.9	0.055	[−21.3, 0.2]
Ezetimibe use (yes vs no)	−6.3	1.7	−3.7	<0.001	[−9.7, −2.9]
PCSK9 inhibitor use (yes vs no)	−12.8	2.3	−5.5	<0.001	[−17.4, −8.2]

a. Sample: N = 943 included (157 excluded due to missingness). Model fit: Residual SE = 22.72; $R^2 = 0.16$; adjusted $R^2 = 0.15$; $F(7, 935) = 25.51$; $p < 0.001$.

b. Medication indicators denote drugs prescribed at hospital discharge from the index admission.

eTable 12. Clinical Events Within 3 Months (Intention-To-Treat Population)

Event	Total (n=1100)	Control (n=551)	Intervention (n=549)
Death	2 (<1%)	1 (<1%)	1 (<1%)
Myocardial infarction	3 (<1%)	0	3 (1%)
Rehospitalisation due to thrombocytopenia	1 (<1%)	0	1 (<1%)
Rehospitalisation due to poor risk-factor control	3 (<1%)	1 (<1%)	2 (<1%)
Rehospitalisation due to symptoms	17 (2%)	10 (2%)	7 (1%)

Data are n (%).

eFigure 1. Operational logic and management indicators of the AIM-CHD

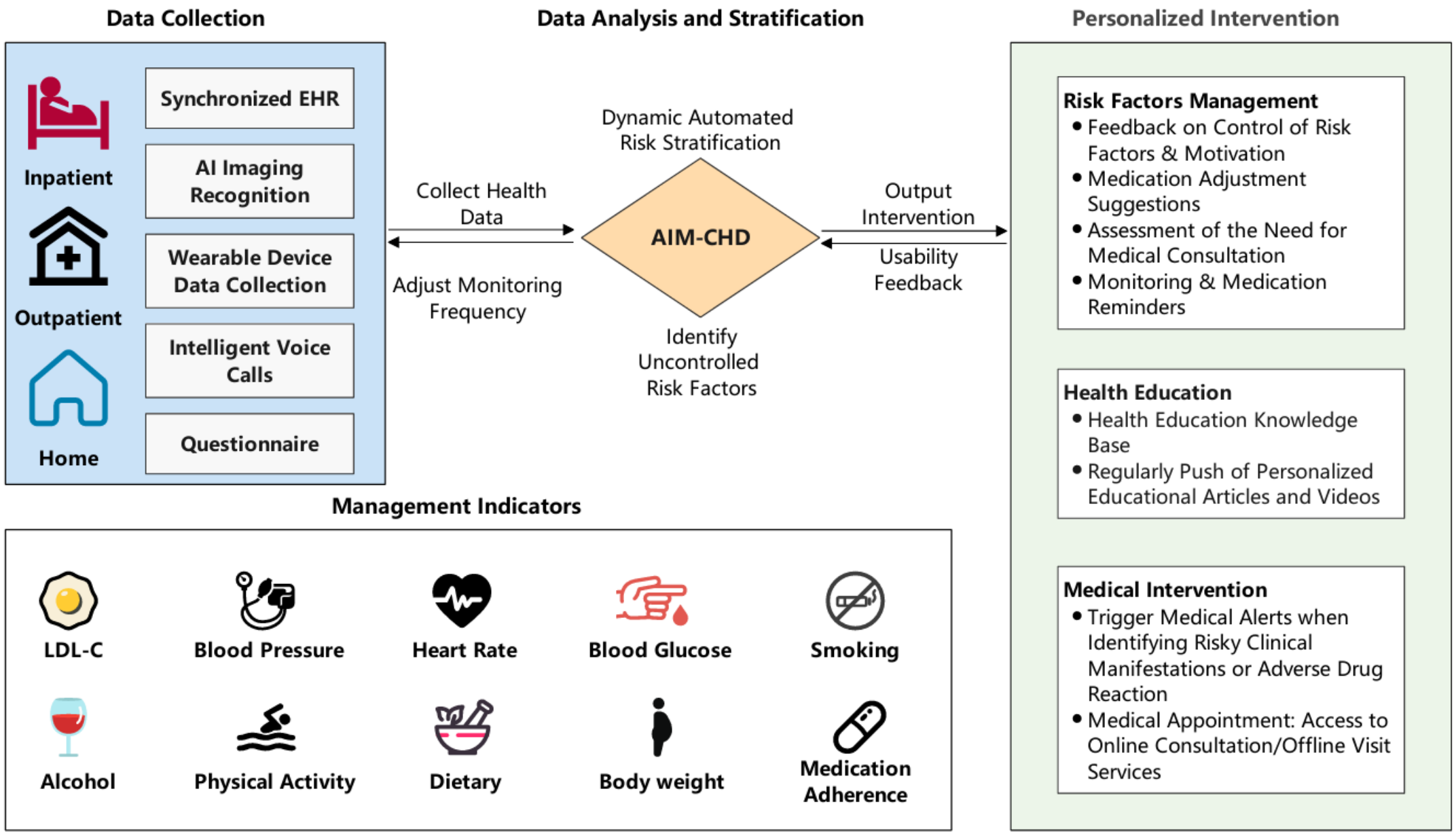

EHR, Electronic Health Record; AI, Artificial Intelligence; LDL-C, Low-Density Lipoprotein Cholesterol; AIM-CHD, AI-enhanced management system for coronary heart disease. Adapted from Ba Z, Zhao S, Liu M, et al. BMJ Open 2025;15:e105597. Licensed under CC BY-NC 4.0 (http://creativecommons.org/licenses/by-nc/4.0/).

eFigure 2. Software Backend

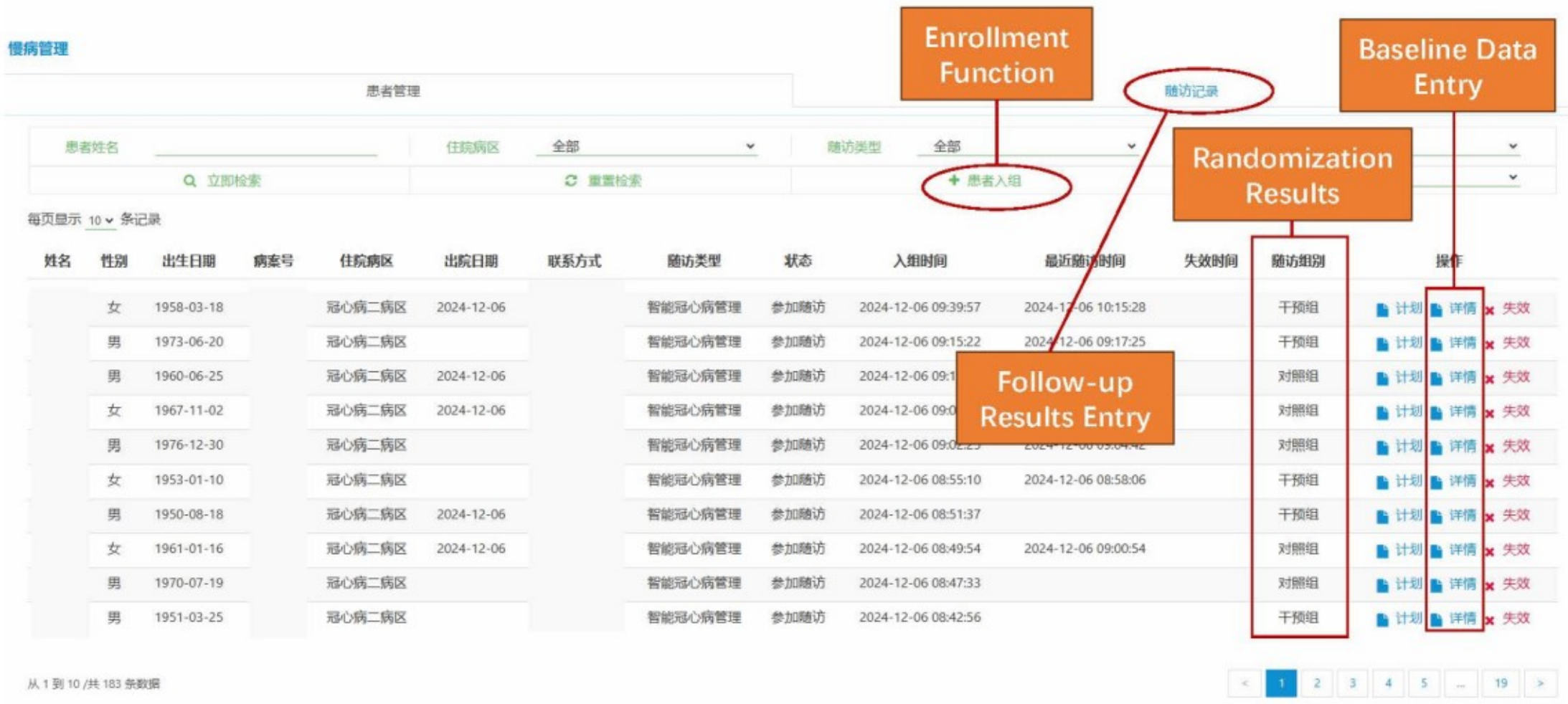

The enrollment and randomization systems are embedded in the AIM-CHD software backend. By entering the patient's hospitalization ID, inpatient data is automatically synchronized, and patients are then randomly assigned to groups. Patients in the intervention group are granted access to the AIM-CHD platform. The backend provides access to baseline and follow-up information for each patient.

eFigure 3. Software Main Interface

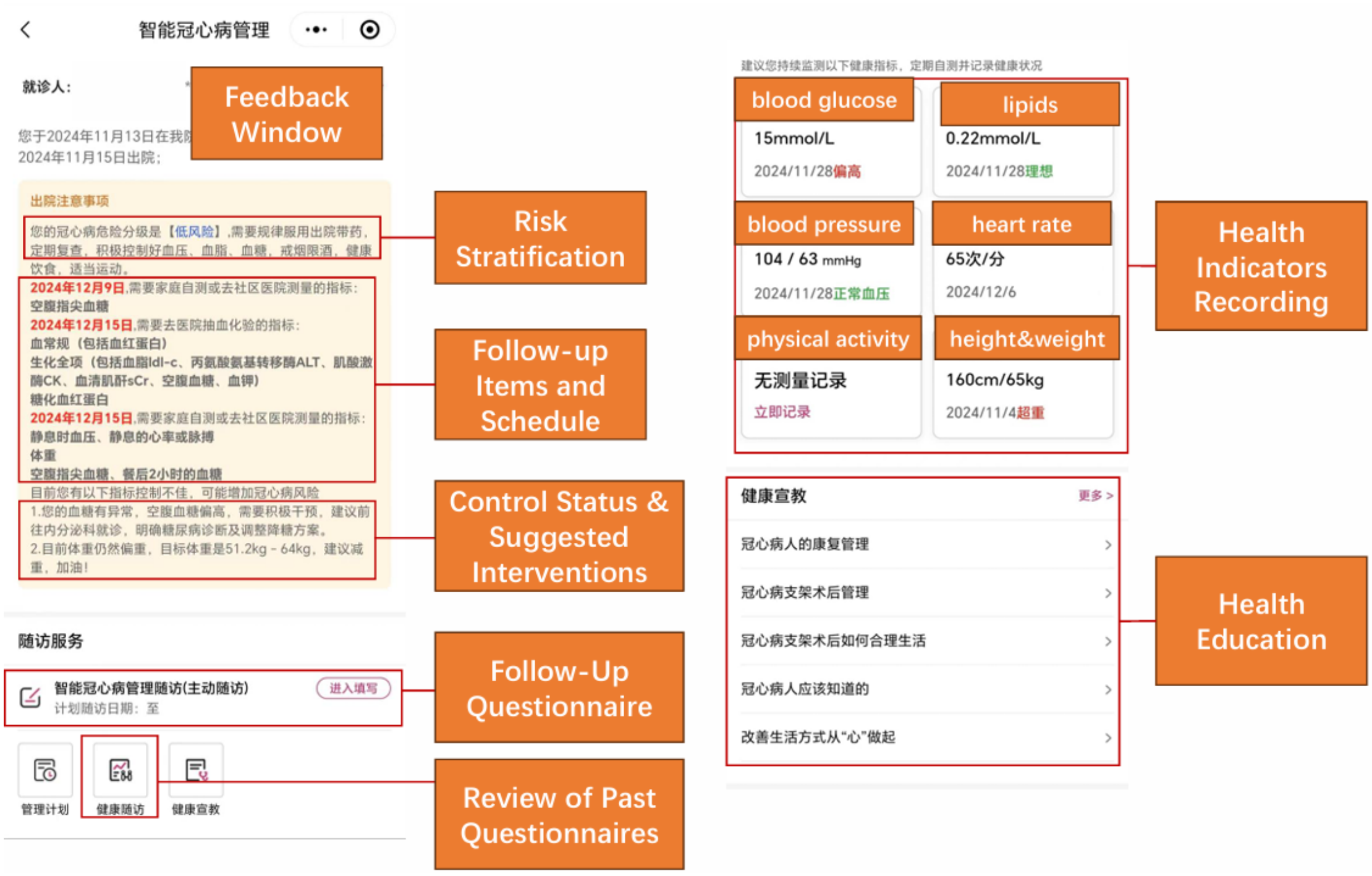

After integrating data from multiple sources, the Feedback Window is updated in real-time. The feedback includes GRACE risk stratification results, next scheduled follow-up indicators, corresponding follow-up times, current abnormal indicators, suggested interventions, and recommendations for medical consultation if needed. Once synchronization with the patient's baseline discharge data, the system generates an initial feedback summary. Patients can use the platform to complete the Proactive Follow-Up Questionnaire and record Health Indicators Recording, receiving real-time feedback. When scheduled follow-up dates approach, the Proactive Follow-Up Questionnaire transitions to a Planned Follow-Up Questionnaire, with reminders sent via WeChat notifications and automated AI voice calls. Patients can access educational videos and articles in the Health Education section.

eFigure 4. Questionnaire Interface

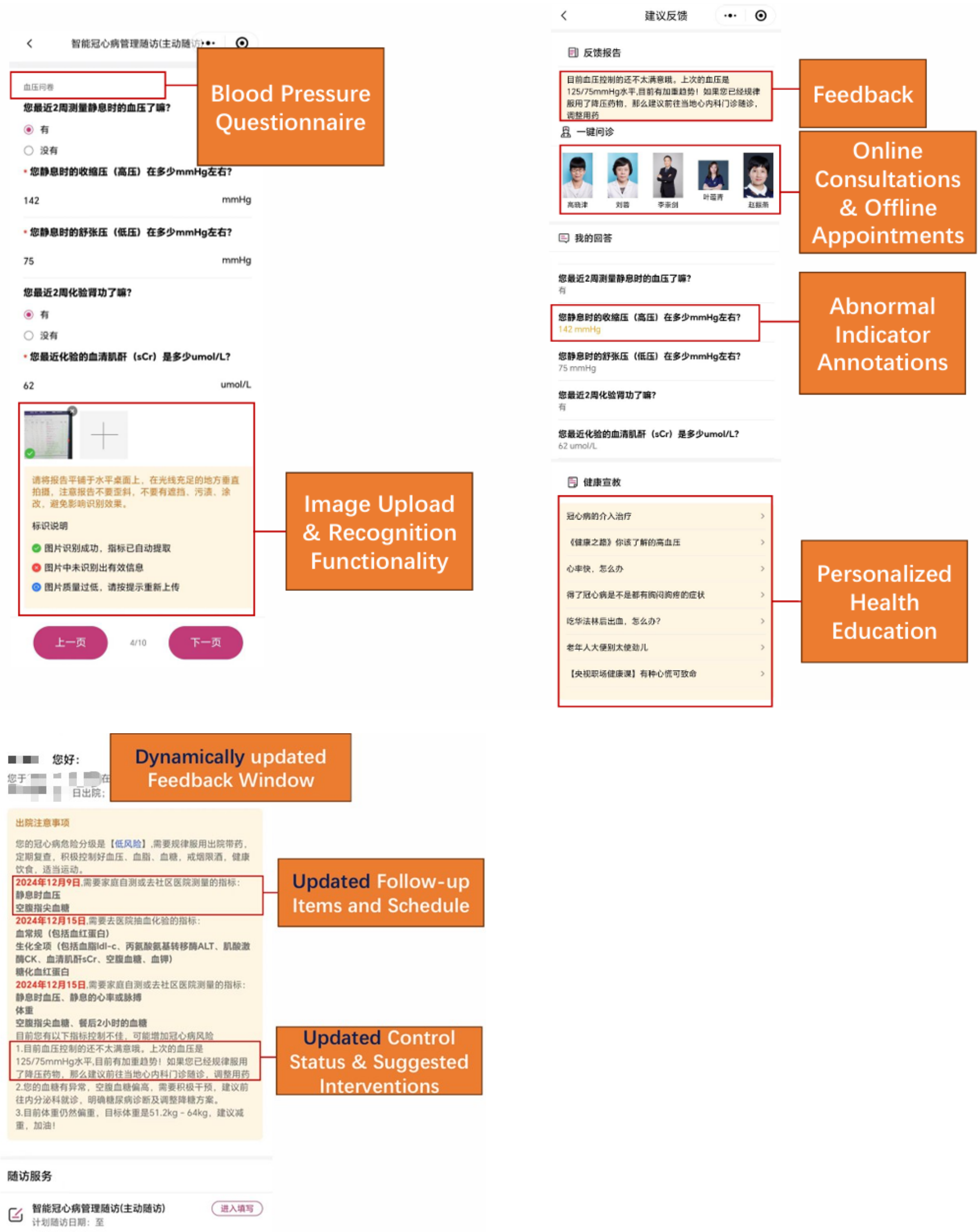

Most questionnaire items are optional, allowing patients to selectively complete sections relevant to their available health data. Additionally, laboratory results can be automatically extracted through image recognition technology. Upon questionnaire submission, feedback is generated immediately, including links to relevant health education videos. If results indicate significant abnormalities, patients are advised to seek medical attention with options for both online consultations and offline clinic appointment bookings. The feedback is also synchronized with the main interface’s Feedback Window. Example: For patients with markedly high systolic blood pressure, the system adjusts the frequency of blood pressure monitoring and advises keep antihypertensive medication while promptly seeking medical consultation.

eFigure 5. Health Indicators Recording

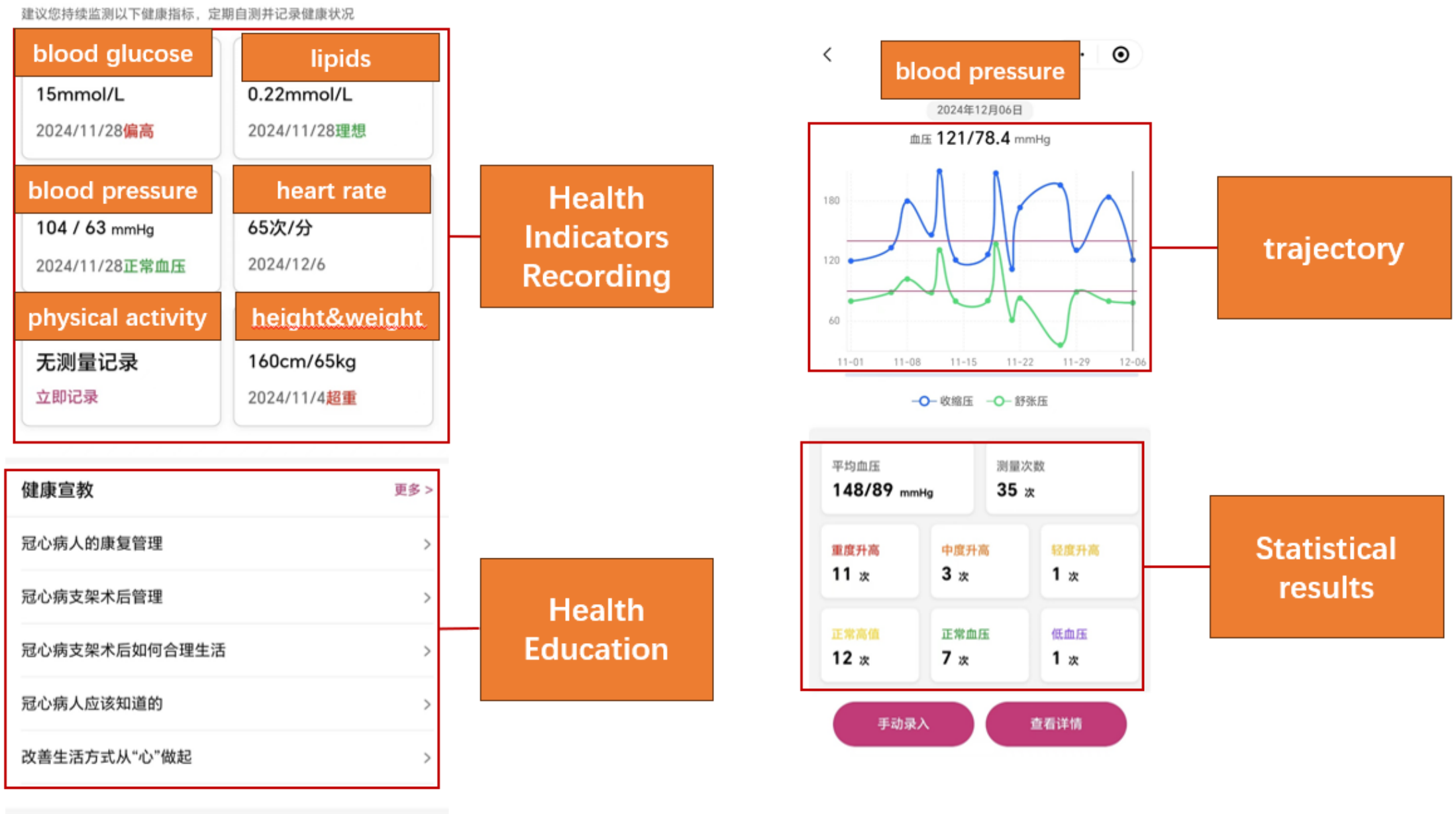

Patients can log their blood glucose, lipids levels, blood pressure, heart rate, physical activity (step count), height, and weight in the Health Indicators Recording section. Data from wearable devices, such as Huawei smartwatches, can be automatically synchronized. After data entry or synchronization, the system evaluates for abnormalities and provides feedback in the Feedback Window in real time.

eFigure 6. Medication Reminders

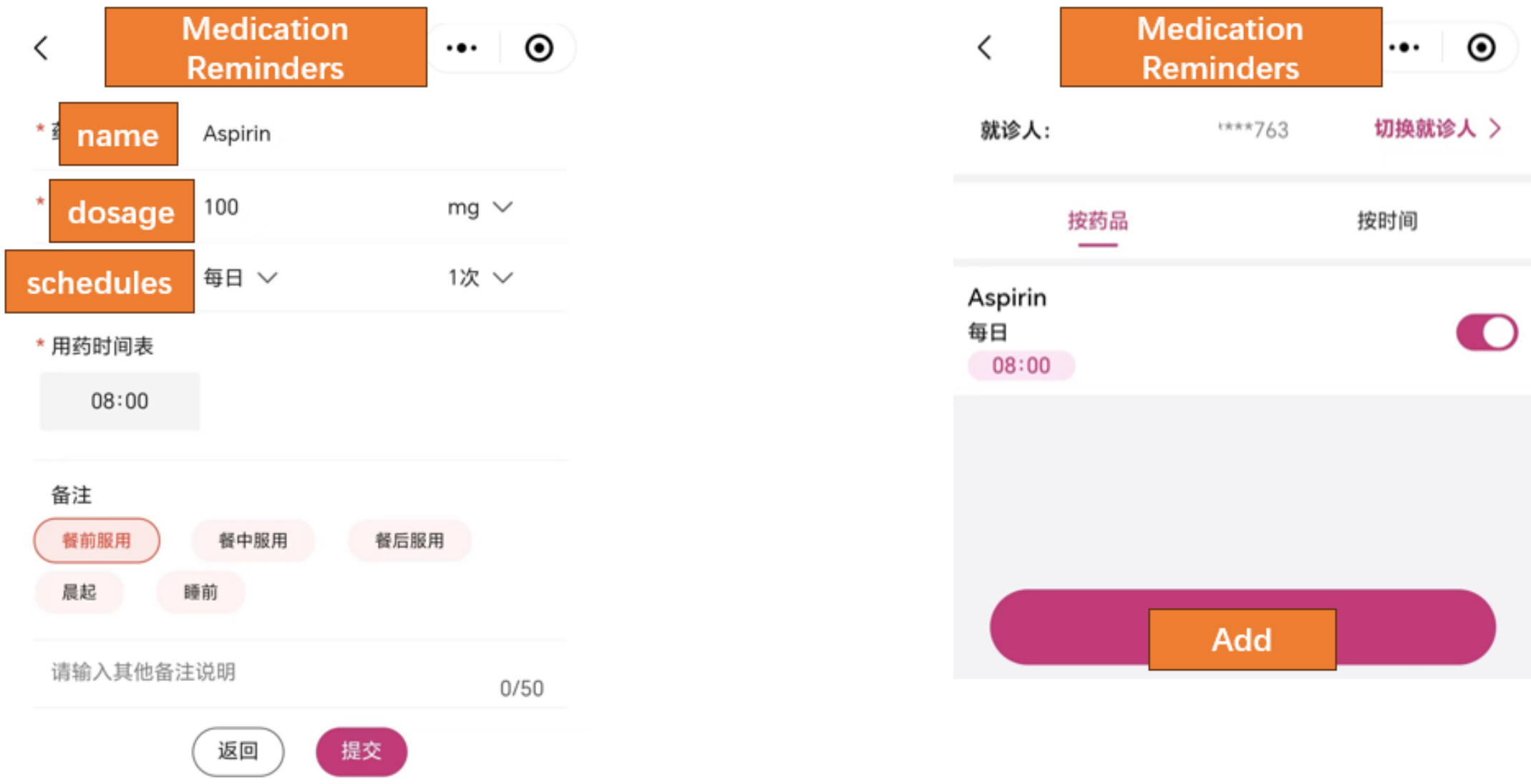

Patients can manually enter medication names, dosages, and schedules. The system then sends timely reminders to ensure medication adherence.

eFigure 7. Health Education Section

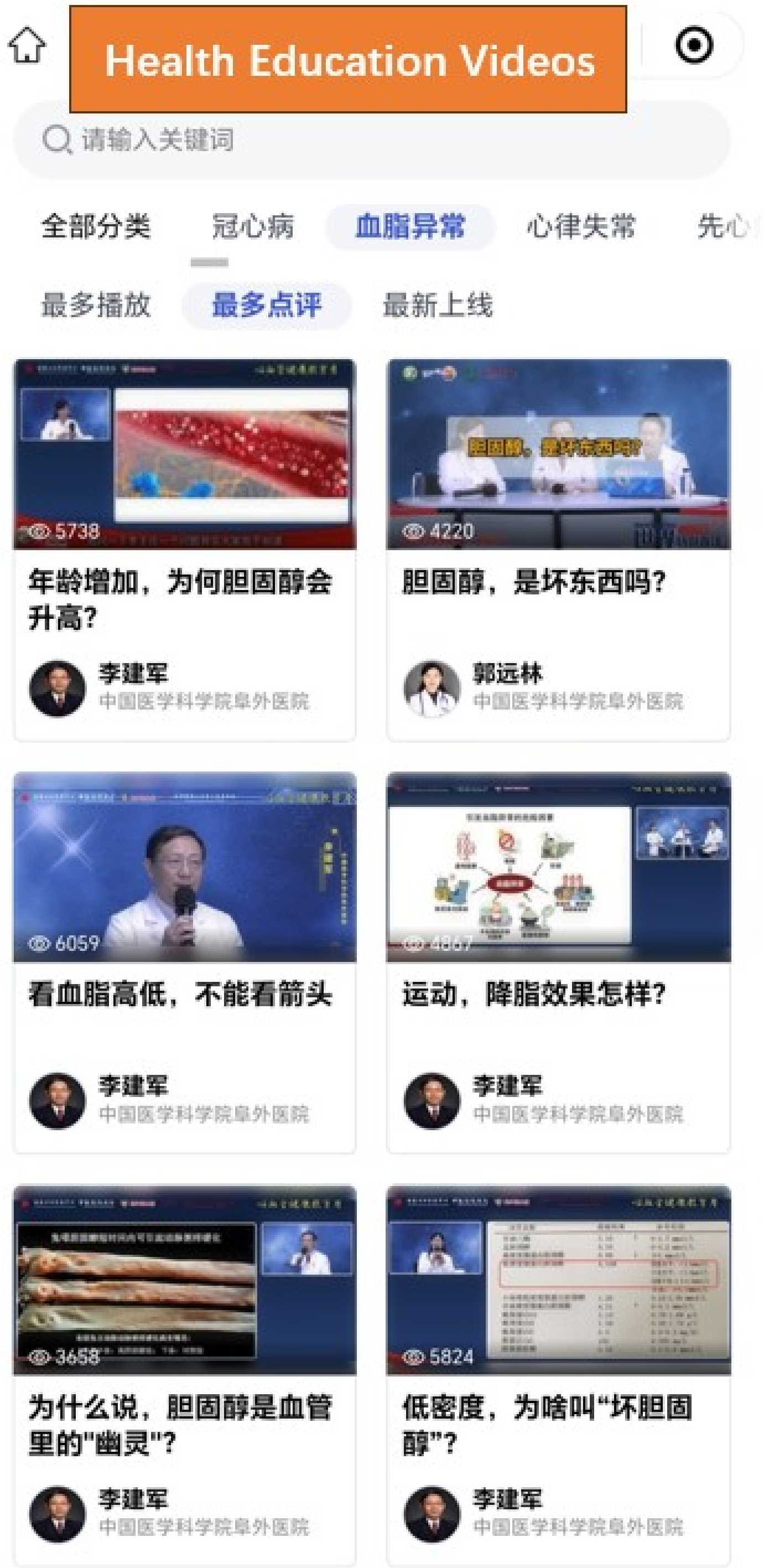

This section offers disease-specific educational videos and articles, which are periodically and individually tailored for each patient to view.

eFigure 8. Subgroup analysis of LDL-C at 3-month

Subgroup	Adjusted mean difference (95% CI)	P value for interaction
**Sex**		.07
Male (n=708)	-3.2 (-6.8 to 0.4)	
Female (n=235)	-4.3 (-10.7 to 2.0)	
**Age (years)**		.87
≤45 (n=95)	-8.0 (-17.9 to 2.0)	
46–60 (n=379)	-2.4 (-7.5 to 2.6)	
>60 (n=469)	-3.0 (-7.4 to 1.4)	
**Education**		.26
Low education (n=350)	-2.3 (-8.1 to 3.5)	
Higher education (n=190)	-3.2 (-9.5 to 3.1)	
Intermediate education (n=403)	-3.9 (-8.3 to 0.6)	
**Ethnicity**		.93
Han Chinese (n=880)	-3.5 (-6.8 to -0.2)	
Non-Han Chinese ethnicities (n=63)	-0.2 (-12.0 to 11.6)	
**Smoking status**		.53
No (n=458)	-2.2 (-6.7 to 2.2)	
Yes (n=485)	-4.5 (-9.0 to -0.0)	
**Risk stratification**		.93
Low risk (n=773)	-3.1 (-6.6 to 0.4)	
High risk (n=28)	-9.2 (-30.7 to 12.3)	
Intermediate risk (n=142)	-3.3 (-11.5 to 5.0)	
**LDL-C at baseline**		.05
LDL-C <1.8 mmol/L (n=427)	-1.0 (-4.7 to 2.6)	
LDL-C ≥1.8 mmol/L (n=516)	-5.8 (-10.4 to -1.2)	

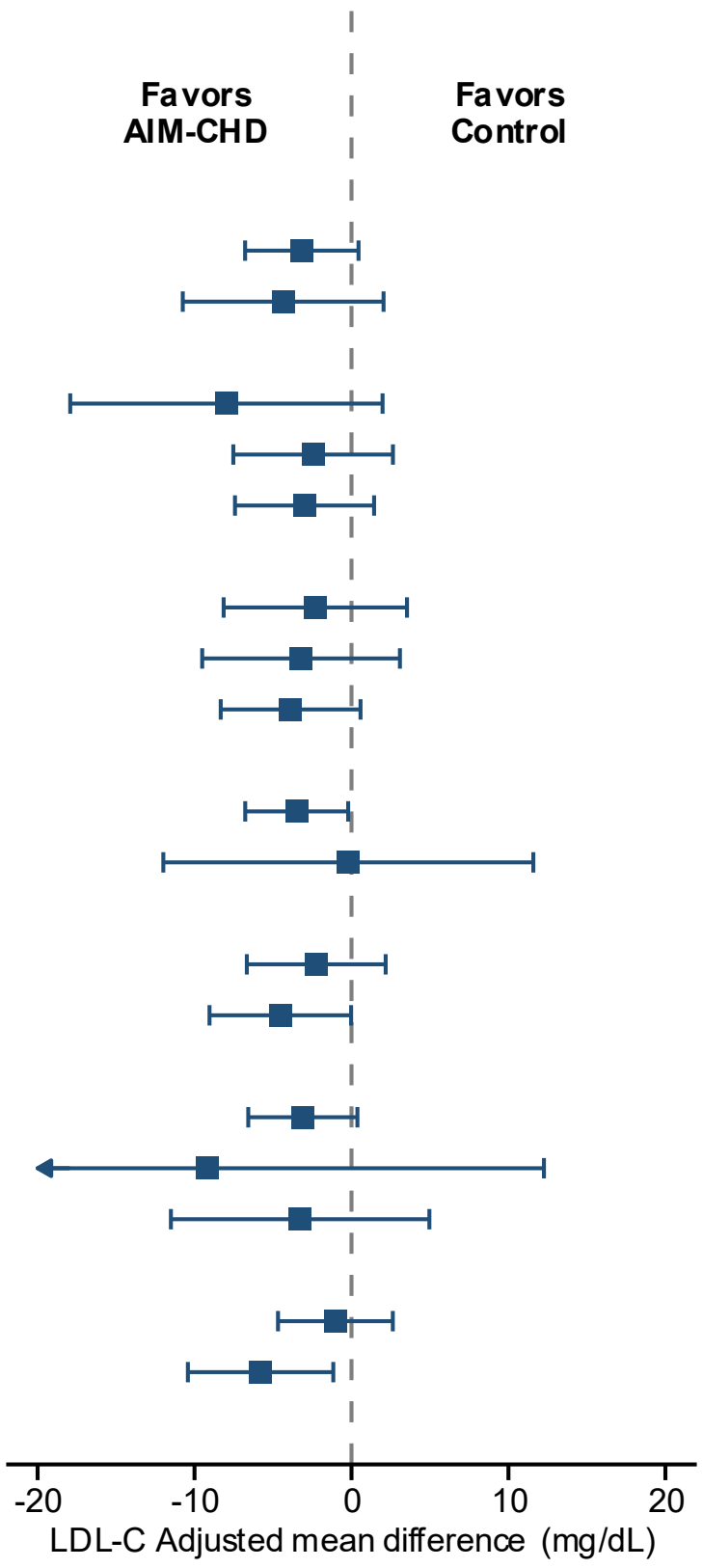

eFigure 9. Subgroup analysis of lipid target attainment at 3-month

Subgroup	Adjusted mean difference (95% CI)	P value for interaction
**Sex**		.53
Male (n=708)	1.1 (1.0 to 1.2)	
Female (n=235)	1.2 (1.0 to 1.5)	
**Age (years)**		.82
<65 (n=607)	1.1 (1.0 to 1.2)	
≥65 (n=336)	1.1 (1.0 to 1.3)	
**Education**		.93
Low education (n=350)	1.1 (1.0 to 1.3)	
Higher education (n=190)	1.1 (1.0 to 1.4)	
Intermediate education (n=403)	1.1 (0.9 to 1.2)	
**Ethnicity**		.92
Han Chinese (n=880)	1.1 (1.0 to 1.2)	
Non-Han Chinese ethnicities (n=63)	1.1 (0.8 to 1.4)	
**Smoking status**		.68
No (n=458)	1.1 (1.0 to 1.3)	
Yes (n=485)	1.1 (1.0 to 1.2)	
**Risk stratification**		.98
Low risk (n=773)	1.1 (1.0 to 1.2)	
High risk (n=28)	1.1 (0.7 to 1.7)	
Intermediate risk (n=142)	1.1 (0.9 to 1.5)	
**LDL-C at baseline**		.40
LDL-C <1.8 mmol/L (n=427)	1.0 (0.9 to 1.1)	
LDL-C ≥1.8 mmol/L (n=516)	1.2 (1.0 to 1.4)	

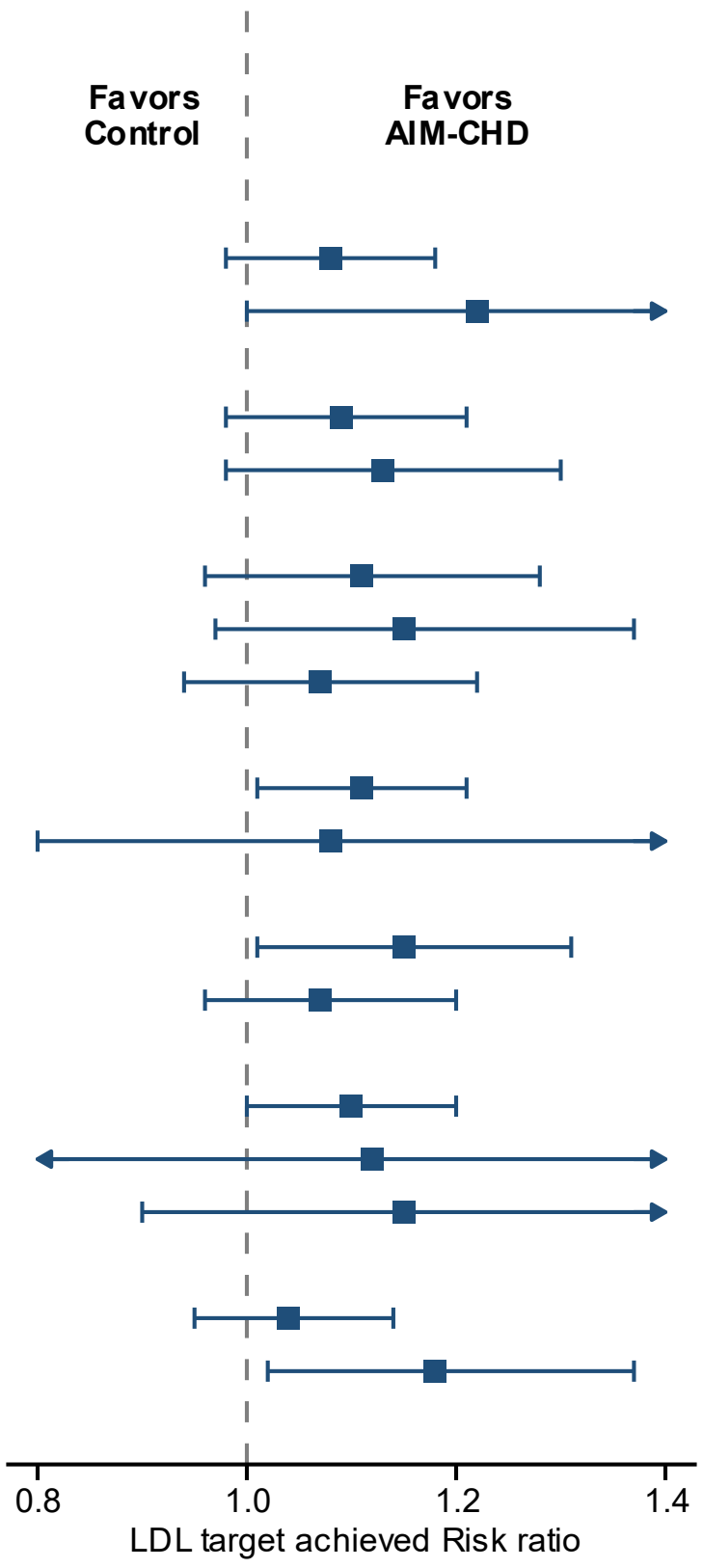

eFigure 10. Subgroup analysis of blood-pressure target attainment at 3-month

Subgroup	Adjusted mean difference (95% CI)	P value for interaction
**Sex**		.40
Male (n=716)	1.4 (1.1 to 1.6)	
Female (n=236)	1.1 (0.8 to 1.5)	
**Age (years)**		.58
<65 (n=627)	1.3 (1.1 to 1.6)	
≥65 (n=325)	1.2 (0.9 to 1.6)	
**Education**		.46
Low education (n=364)	1.3 (0.9 to 1.8)	
Higher education (n=188)	1.6 (1.2 to 2.2)	
Intermediate education (n=400)	1.1 (0.9 to 1.4)	
**Ethnicity**		.98
Han Chinese (n=891)	1.3 (1.1 to 1.5)	
Non-Han Chinese ethnicities (n=61)	1.2 (0.6 to 2.4)	
**Smoking status**		.68
No (n=451)	1.2 (1.0 to 1.6)	
Yes (n=501)	1.3 (1.1 to 1.6)	
**Risk stratification**		.97
Low risk (n=788)	1.3 (1.1 to 1.6)	
High risk (n=21)	1.1 (0.4 to 3.4)	
Intermediate risk (n=143)	1.3 (0.8 to 1.9)	
**LDL-C at baseline**		.92
LDL-C <1.8 mmol/L (n=422)	1.3 (1.0 to 1.6)	
LDL-C ≥1.8 mmol/L (n=530)	1.3 (1.0 to 1.6)	
**Hypertension**		.81
No (n=300)	1.4 (1.1 to 1.7)	
Yes (n=652)	1.2 (1.0 to 1.6)	

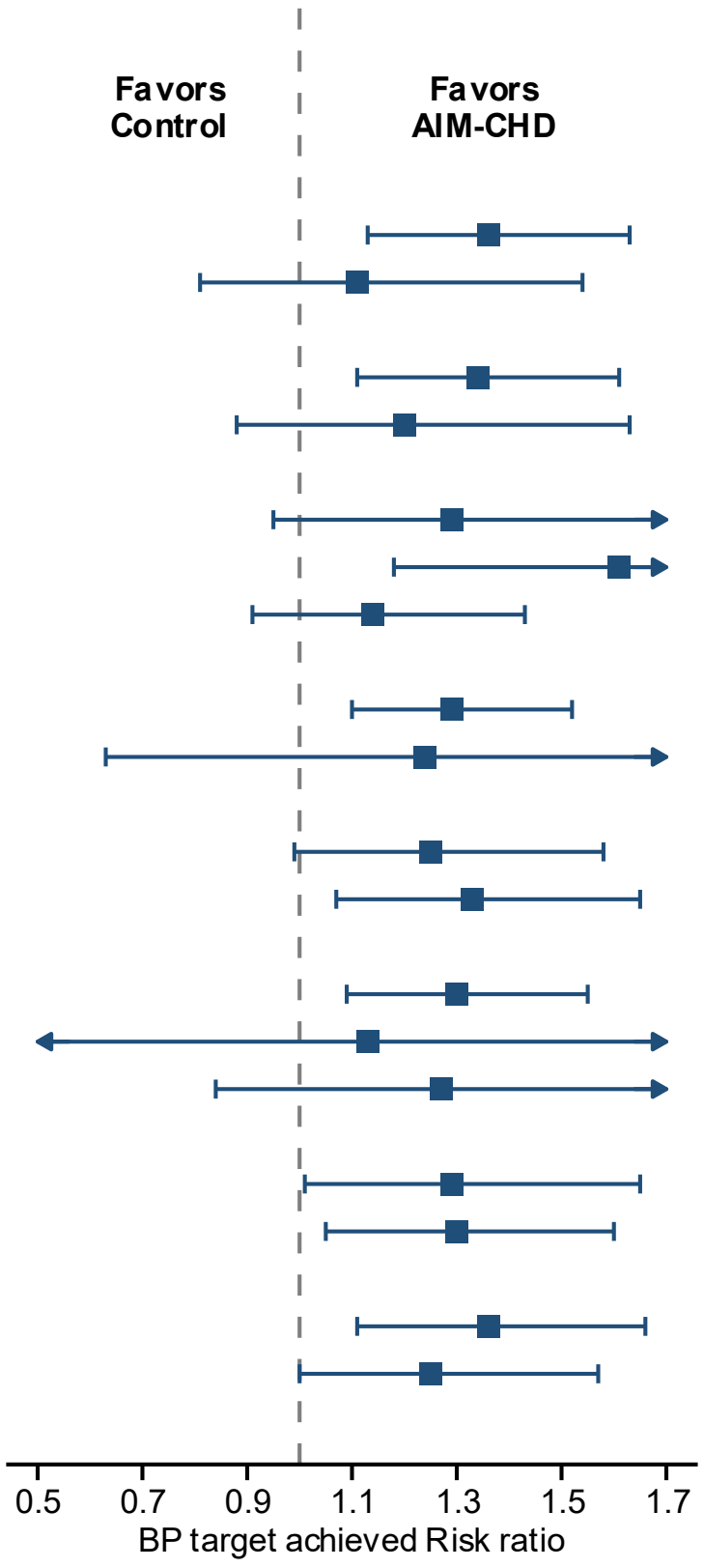

eFigure 11. Subgroup analysis of Non-smoking status at 3-month

Subgroup	Adjusted mean difference (95% CI)	P value for interaction
**Sex**		.69
Male (n=740)	1.1 (1.0 to 1.1)	
Female (n=234)	1.0 (1.0 to 1.1)	
**Age (years)**		.80
<65 (n=638)	1.1 (1.0 to 1.1)	
≥65 (n=336)	1.0 (1.0 to 1.1)	
**Education**		.95
Low education (n=366)	1.0 (1.0 to 1.1)	
Higher education (n=190)	1.0 (0.9 to 1.1)	
Intermediate education (n=418)	1.1 (1.0 to 1.2)	
**Ethnicity**		.69
Han Chinese (n=904)	1.1 (1.0 to 1.1)	
Non-Han Chinese ethnicities (n=70)	0.9 (0.7 to 1.2)	
**Smoking status**		.59
Yes (n=515)	1.1 (1.0 to 1.2)	
**Risk stratification**		.98
Low risk (n=804)	1.0 (1.0 to 1.1)	
Intermediate risk (n=145)	1.1 (1.0 to 1.2)	
**LDL-C at baseline**		.68
LDL-C <1.8 mmol/L (n=427)	1.0 (1.0 to 1.1)	
LDL-C ≥1.8 mmol/L (n=547)	1.1 (1.0 to 1.2)	

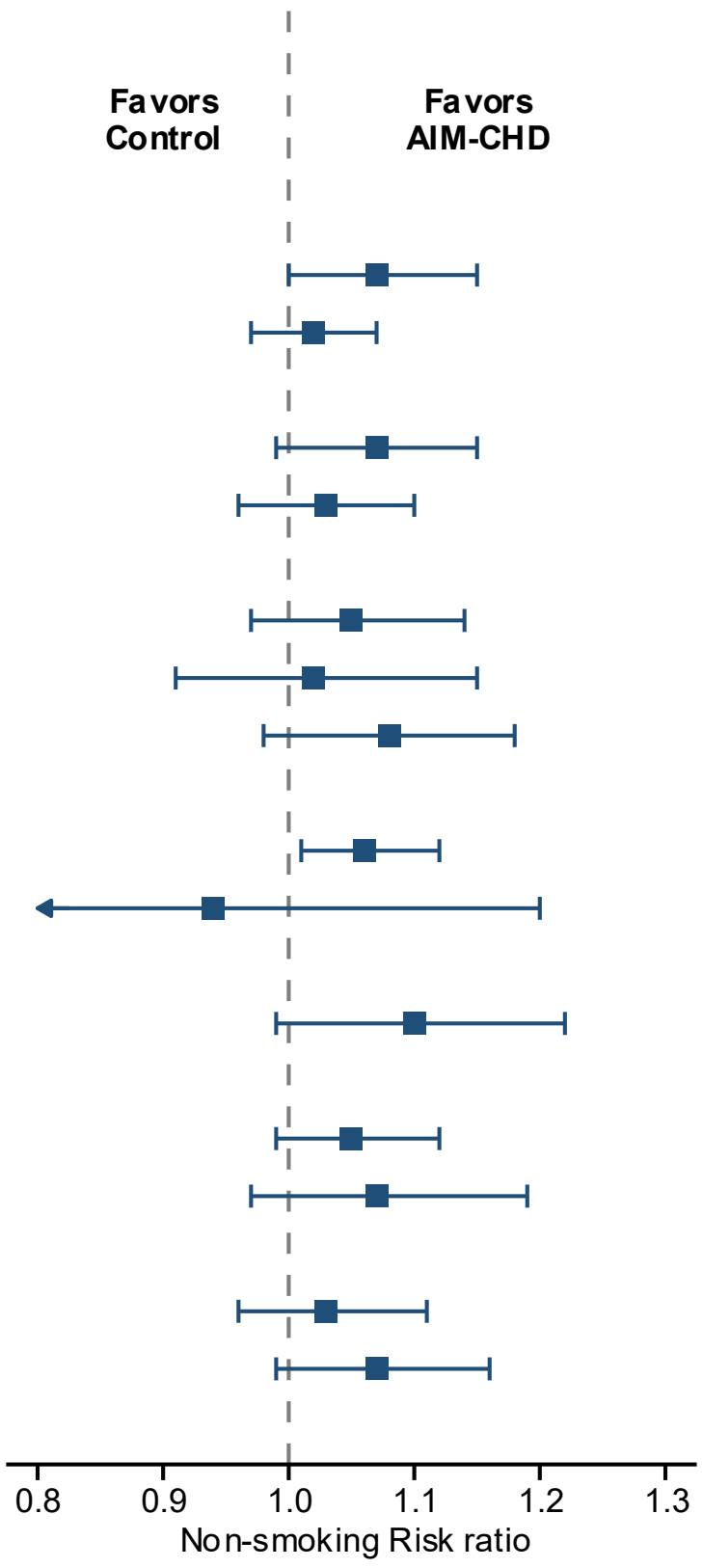

eFigure 12. Subgroup analysis of HbA1c at 3-month

Subgroup	Adjusted mean difference (95% CI)	P value for interaction
**Sex**		.94
Male (n=311)	0.0 (-0.2 to 0.2)	
Female (n=99)	-0.4 (-0.9 to 0.0)	
**Age (years)**		.08
≤45 (n=43)	-0.5 (-1.1 to 0.1)	
46–60 (n=162)	0.2 (-0.2 to 0.5)	
>60 (n=205)	-0.2 (-0.5 to 0.1)	
**Education**		.24
Low education (n=142)	-0.3 (-0.7 to 0.0)	
Higher education (n=85)	-0.1 (-0.5 to 0.3)	
Intermediate education (n=183)	0.1 (-0.2 to 0.4)	
**Ethnicity**		.89
Han Chinese (n=387)	-0.1 (-0.3 to 0.1)	
Non-Han Chinese ethnicities (n=23)	-0.4 (-1.3 to 0.5)	
**Smoking status**		.60
No (n=206)	-0.1 (-0.4 to 0.2)	
Yes (n=204)	-0.1 (-0.3 to 0.2)	
**Risk stratification**		.23
Low risk (n=344)	-0.0 (-0.2 to 0.2)	
High risk (n=13)	-1.1 (-1.9 to -0.4)	
Intermediate risk (n=53)	-0.3 (-0.8 to 0.2)	
**LDL-C at baseline**		.71
LDL-C <1.8 mmol/L (n=204)	-0.0 (-0.3 to 0.2)	
LDL-C ≥1.8 mmol/L (n=206)	-0.1 (-0.4 to 0.2)	
**Diabetes mellitus**		.08
No (n=200)	-0.1 (-0.2 to 0.1)	
Yes (n=210)	0.0 (-0.3 to 0.3)	

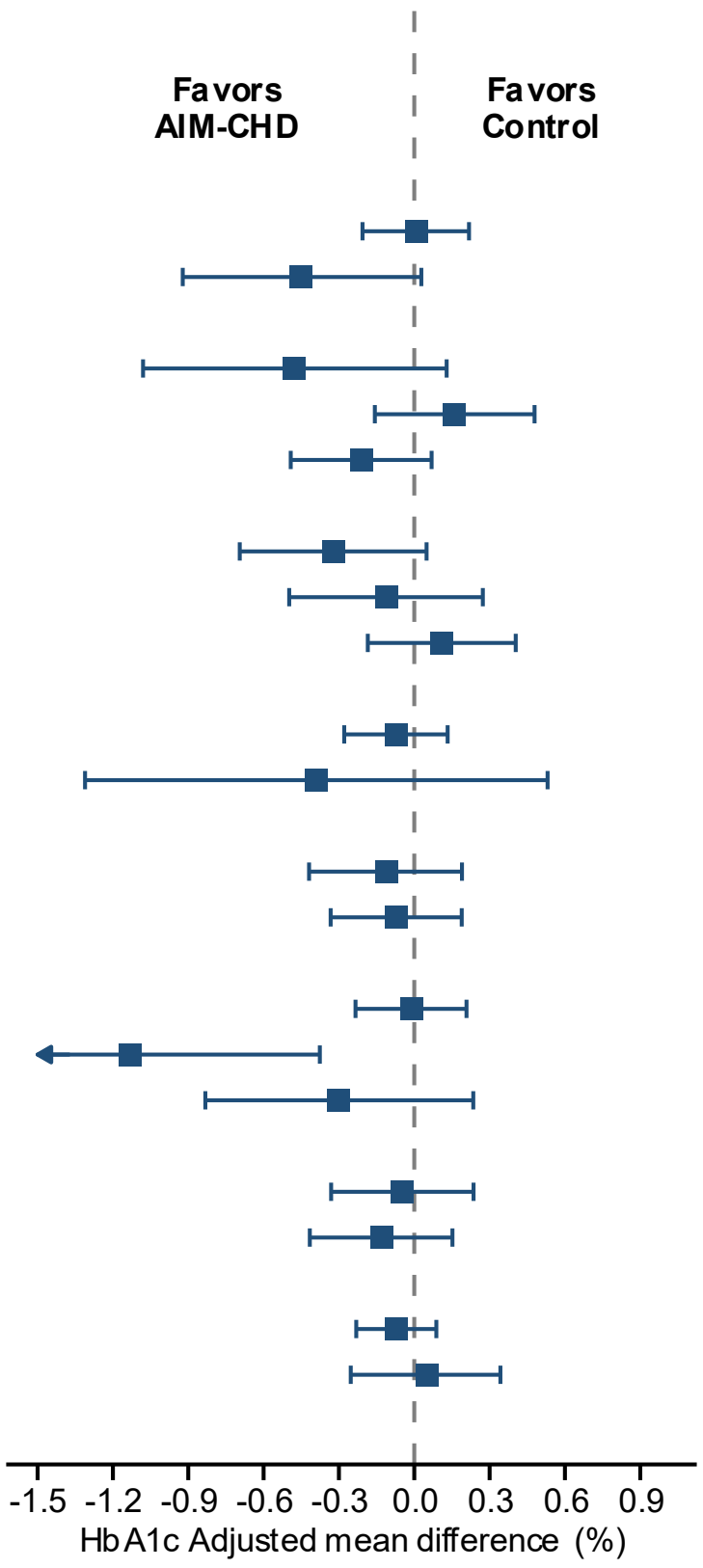

eFigure 13. Subgroup analysis of BMI at 3-month

Subgroup	Adjusted mean difference (95% CI)	P value for interaction
**Sex**		.53
Male (n=716)	-0.4 (-0.9 to 0.1)	
Female (n=234)	-0.6 (-1.5 to 0.4)	
**Age (years)**		.95
≤45 (n=107)	-0.1 (-1.4 to 1.3)	
46–60 (n=387)	-0.2 (-0.8 to 0.5)	
>60 (n=456)	-0.6 (-1.2 to -0.0)	
**Education**		.57
Low education (n=346)	-0.8 (-1.5 to -0.1)	
Higher education (n=194)	0.4 (-0.6 to 1.3)	
Intermediate education (n=410)	-0.6 (-1.3 to 0.0)	
**Ethnicity**		.78
Han Chinese (n=884)	-0.4 (-0.9 to -0.0)	
Non-Han Chinese ethnicities (n=66)	-1.0 (-2.6 to 0.6)	
**Smoking status**		.14
No (n=454)	-0.4 (-1.1 to 0.2)	
Yes (n=496)	-0.4 (-1.0 to 0.1)	
**Risk stratification**		.35
Low risk (n=791)	-0.5 (-0.9 to -0.0)	
High risk (n=23)	-0.1 (-4.5 to 4.3)	
Intermediate risk (n=136)	-0.3 (-1.4 to 0.9)	
**LDL-C at baseline**		.13
LDL-C <1.8 mmol/L (n=423)	-0.1 (-0.8 to 0.6)	
LDL-C ≥1.8 mmol/L (n=527)	-0.8 (-1.3 to -0.2)	

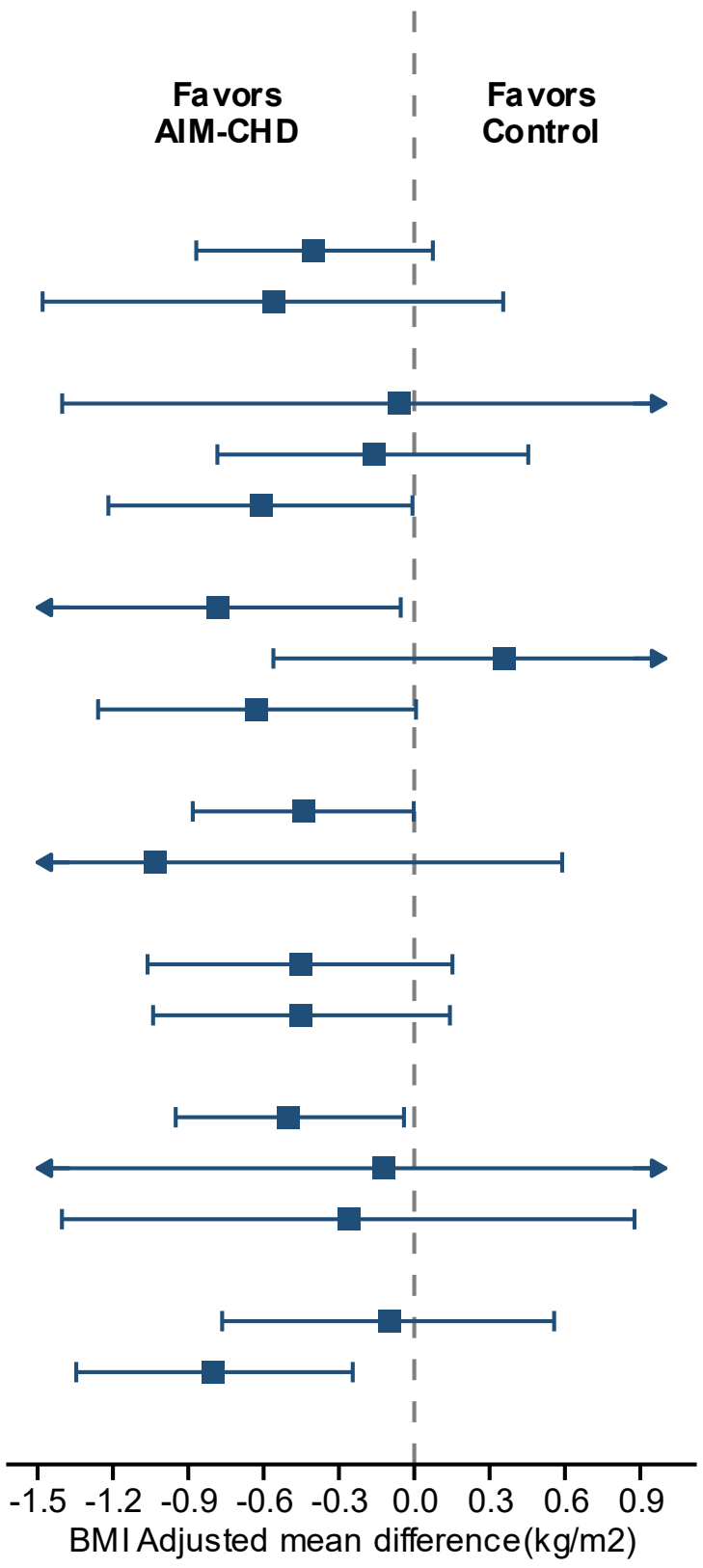

eFigure 14. Subgroup analysis of medication adherence at 3-month

Subgroup	Adjusted mean difference (95% CI)	P value for interaction
**Sex**		.86
Male (n=725)	1.0 (1.0 to 1.1)	
Female (n=243)	1.0 (1.0 to 1.1)	
**Age (years)**		.92
<65 (n=633)	1.0 (1.0 to 1.1)	
≥65 (n=335)	1.0 (1.0 to 1.1)	
**Education**		.92
Low education (n=363)	1.0 (0.9 to 1.0)	
Higher education (n=189)	1.0 (0.9 to 1.1)	
Intermediate education (n=416)	1.1 (1.0 to 1.1)	
**Ethnicity**		.91
Han Chinese (n=896)	1.0 (1.0 to 1.1)	
Non-Han Chinese ethnicities (n=72)	1.0 (0.9 to 1.1)	
**Smoking status**		.97
No (n=467)	1.0 (1.0 to 1.1)	
Yes (n=501)	1.0 (1.0 to 1.1)	
**Risk stratification**		.94
Low risk (n=802)	1.0 (1.0 to 1.1)	
High risk (n=23)	0.9 (0.8 to 1.1)	
Intermediate risk (n=143)	1.1 (1.0 to 1.2)	
**LDL-C at baseline**		.86
LDL-C <1.8 mmol/L (n=425)	1.0 (1.0 to 1.1)	
LDL-C ≥1.8 mmol/L (n=543)	1.0 (1.0 to 1.1)	

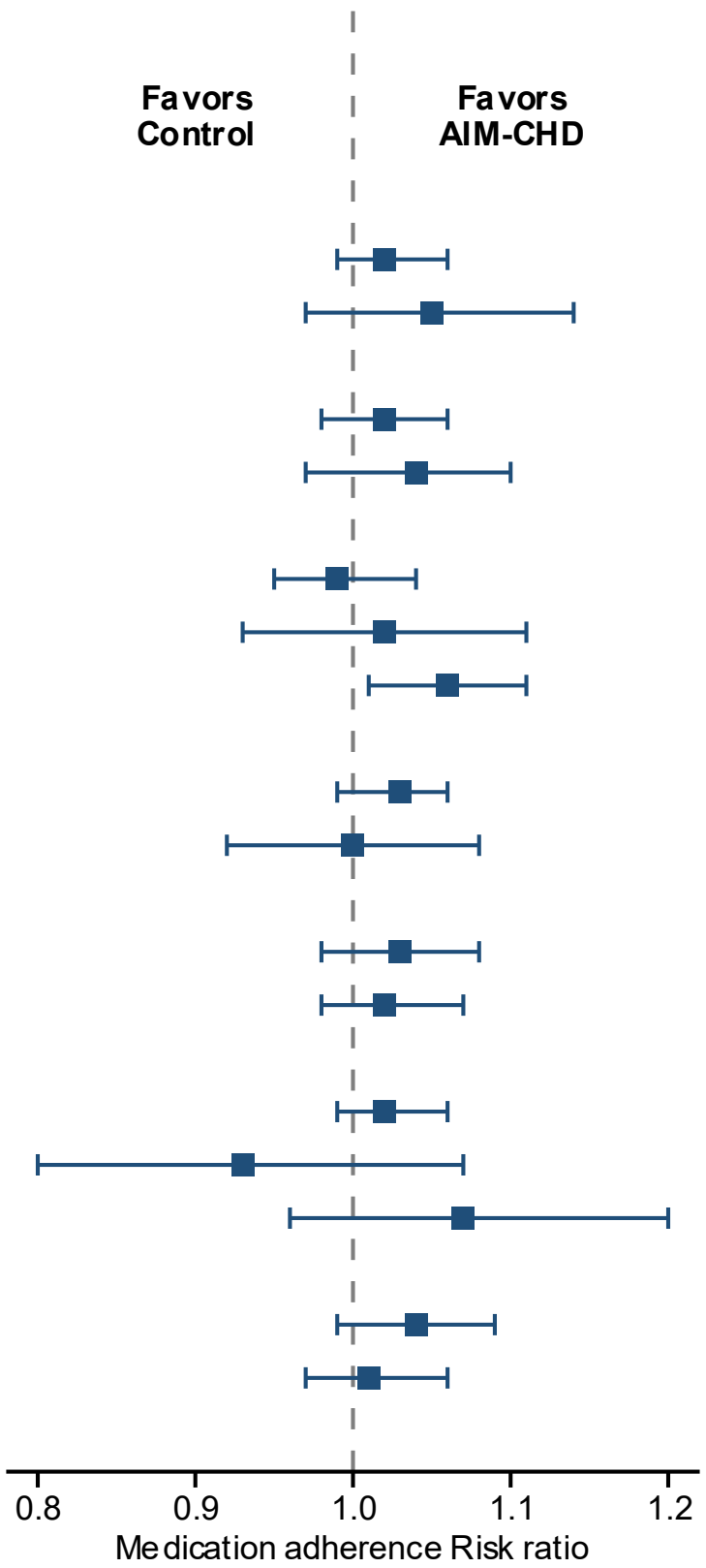

Study Title: Development and Clinical Evaluation of an Artificial Intelligence–Based Stratified Management Platform for Coronary Heart Disease

Study Protocol

Version: V1.2

Version Date: October 22, 2024

Funding Source: Clinical Research Fund of Fuwai Hospital, Chinese Academy of Medical Sciences (the National High Level Hospital Clinical Research Funding)

Principal Investigator: Dr. Xiaojin Gao

Statistical Unit: Department of Medical Research and Biometrics Center, National Center for Cardiovascular Diseases, China

Table of Contents

Protocol Summary 4

1. Background and Rationale 6

2. Study Objectives 7

3. Methods 7

    3.1 Overall Study Design 7

    3.2 Participant Selection 8

    3.3 Interventions 9

    3.4 Study Flow 11

    3.5 Criteria for Study Suspension or Termination 14

    3.6 Outcome Measures 15

    3.7 Measures to Minimise Bias 15

4. Statistical Considerations 16

    4.1 Sample Size Estimation 16

    4.2 Statistical Analysis Plan 17

5. Confidentiality 18

6. Quality Assurance 19

    6.1 Case Report Form (CRF) and Tool Development 19

    6.2 Patient Eligibility 19

    6.3 Data Entry 19

    6.4 Data Cleaning 20

    6.5 Data Analysis 20

7. Ethical Principles 20

7.1 Ethical Approval 20

7.2 Informed Consent 20

7.3 Risk－Benefit Assessment 21

7.4 Conflict of Interest 21

7.5 Participant Protection Measures 21

8. Project Risk Assessment and Contingency Plan 22

9. Protocol Deviations and Amendments 23

10. Study Organisation and Responsibilities 23

11. References 23

Protocol Summary

Study Title	Development and Clinical Evaluation of an Artificial Intelligence–Based Stratified Management Platform for Coronary Heart Disease
Objective	To conduct a randomized controlled clinical trial to evaluate the clinical value of a generative AI–enhanced system based on an innovative long-term management model for coronary heart disease. The study aims to verify whether this system can improve control of key risk factors (e.g., LDL-C) and reduce cardiac events within 3 months after discharge.
Study Design	Prospective, single-center, open-label, randomized, controlled clinical trial
Study Population	Patients with coronary heart disease hospitalized at Fuwai Hospital
Interventions	Intervention group: Post-discharge management using the AI-based stratified management platform for coronary heart disease plus routine standard of care.<br>Control group: Post-discharge management using the routine standard of care.
Inclusion Criteria	1. Patients aged 18–85 years with diagnosed coronary heart disease;<br>2. The patient or a close family member is able to operate a smartphone and application (App);<br>3. Willingness to participate in the study and provision of written informed consent.
Exclusion Criteria	1. Severe cognitive impairment;<br>2. Advanced malignant tumor;<br>3. Expected survival of less than 3 months;<br>4. Severe multi-organ failure;<br>5. Refusal to sign informed consent.
Primary Endpoint	Low-density lipoprotein cholesterol (LDL-C) level at 3 months after discharge
Secondary Endpoints	1. Key secondary endpoint: Proportion of participants achieving LDL-C target (<1.8 mmol/L) at 3 months after discharge.<br>2. Other secondary endpoints:<br>(1) Blood pressure target achievement rate at 3 months after discharge;<br>(2) Body mass index (BMI) at 3 months after discharge;<br>(3) Smoking rate at 3 months after discharge;<br>(4) Glycated hemoglobin (HbA1c) level at 3 months after discharge;<br>(5) Composite cardiovascular endpoint at 3 months after discharge (defined as the composite of all-cause death, nonfatal myocardial infarction, stroke, or rehospitalization);<br>(6) Adherence to antiplatelet and statin therapy at 3 months after discharge (defined as self-reported medication intake ≥80% of the days in the past month).
Study Period	September 2024 – May 2025
Follow-up Visit	3 months post-procedure/discharge

Sample Size	Total: 1,100 participants<br>• Intervention group: 550<br>• Control group: 550
Sample Size Calculation	The trial is a prospective, single-centre, randomised controlled superiority study. Sample size was determined based on the primary endpoint and expected treatment effect. Based on recent studies of Chinese patients with coronary heart disease under secondary prevention (Cardiovasc Diabetol. 2022–2024), the mean LDL-C ranges from 2.1–2.7 mmol/L (SD 0.80–0.95 mmol/L). Assuming the intervention group achieves a 0.2 mmol/L (≈10%) lower mean LDL-C than the control group at 3 months, with SD 0.95 mmol/L, a superiority test (one-sided α=0.025, power (1–β) = 0.90) using PASS v15.0 yielded a required sample size of 950. Allowing for a 10% attrition rate, the final target is 1,100 participants.
Statistical Evaluation	Analyses will follow the intention-to-treat principle. Continuous outcomes will be analysed using analysis of covariance (ANCOVA) adjusted for baseline values, and binary outcomes with modified Poisson regression to estimate risk ratios (RRs) and 95% confidence intervals (CIs). The composite cardiovascular endpoint will be assessed using Kaplan–Meier curves and Cox proportional-hazards models to estimate hazard ratios (HRs). Missing data will be addressed using multiple imputation (m=20), with sensitivity analyses including complete-case, per-protocol, Δ-tipping-point, IPCW, and best/worst-case analyses. Prespecified subgroup analyses (sex, age, smoking status, baseline LDL-C, and risk stratification) will test for effect modification. All analyses will be performed using R (version 4.4.1). An independent Data Monitoring Committee (DMC) will oversee trial conduct and safety.

1. Background and Rationale

According to the *China Cardiovascular Health and Disease Report 2023*, an estimated 11.39 million individuals in China are living with coronary heart disease (CHD), and this number continues to rise annually. The total hospitalization expenditure for CHD has exceeded 116.9 billion yuan, posing a substantial socioeconomic burden on the healthcare system [1]. As a chronic cardiovascular condition, CHD requires long-term, individualized, and precise management. However, the major bottleneck in achieving such sustained management lies in the limited availability of healthcare resources. For more than 11 million CHD patients in China, implementing long-term, detailed follow-up under the traditional healthcare model would demand enormous financial investment and a vast number of medical professionals. The rapid development and increasing maturity of artificial intelligence (AI) technologies may fundamentally transform this situation.

Both national and international cardiovascular prevention guidelines have emphasized the importance of secondary prevention in CHD [2–5]. Nevertheless, findings from the PURE study revealed that the adoption of healthy lifestyles and secondary prevention pharmacotherapies remains suboptimal across countries with varying income levels, particularly in low- and middle-income regions [6]. More than 40% of patients mistakenly believe that in-hospital treatment has cured their disease and that no further attention to healthy behaviors is necessary. Additional evidence indicates that post-discharge medication adherence is often poor and risk factor control inadequate [7]. Given the enormous patient population, traditional management strategies are constrained by their dependence on intensive healthcare resources. Consequently, researchers worldwide have begun exploring mobile and digital technologies for long-term patient management [8–12]. Studies have shown that interventions delivered via mobile devices and software—such as telephone follow-up, text messaging, and mHealth applications—can improve lifestyle behaviors, risk-factor control, and medication adherence [13–18]; however, some trials have reported inconclusive or inconsistent effects [19–21]. To date, no intelligent management platform has been shown to significantly improve clinical endpoints, which may be attributable to insufficient AI sophistication, narrow intervention content, or small sample sizes [21].

In 2022, our research team received the inaugural AI and Informatics Application Fund from Fuwai Hospital. Building upon the "Palm Fuwai Hospital" mobile application, we developed

an AI-driven, long-term post-discharge management platform for CHD patients, which has been registered with the National Copyright Administration of China [22]. Our preliminary findings demonstrated that intelligent precision management can markedly reduce healthcare resource utilization while providing an AI-based, patient-friendly system capable of automated risk stratification, tiered management, and sustained self-care guidance for patients with premature CHD. The present project aims to conduct a randomized controlled clinical trial to validate the clinical effectiveness and utility of this platform.

2. Study Objectives

This study aims to conduct a prospective, randomized, controlled clinical trial to evaluate the clinical utility of a generative artificial intelligence (AI)–based system designed for innovative long-term management of coronary heart disease (CHD). The primary objective is to determine whether the AI-enhanced management platform can improve the control of low-density lipoprotein cholesterol (LDL-C) and other modifiable risk factors after 3 months of follow-up, as well as to explore its potential to reduce the incidence of cardiac events.

3. Methods

3.1 Overall Study Design

This is a prospective, single-center, open-label, randomized controlled trial.
A total of 1,100 participants with CHD will be consecutively enrolled from the Coronary Heart Disease Center of Fuwai Hospital, Chinese Academy of Medical Sciences, prior to hospital discharge. Participants will be randomly assigned in a 1:1 ratio to one of two groups:1) Intervention group (n = 550): Participants will receive post-discharge management through the AI-based stratified management platform for CHD. 2) Control group (n = 550): Participants will receive standard post-discharge management according to usual care practices. Both groups will be followed for 3 months. Key outcomes—including LDL-C levels, control of other cardiovascular risk factors, and incidence of cardiovascular events—will be assessed and recorded throughout the follow-up period.

3.2 Participant Selection

3.2.1 Inclusion Criteria

Participants must meet all of the following criteria to be eligible for enrollment:

a. Adults aged 18 to 85 years with a confirmed diagnosis of coronary heart disease (CHD);

b. The participant or a close family member is capable of using a smartphone and mobile application (App);

c. The participant agrees to join the study and provides written informed consent.

3.2.2 Exclusion Criteria

Participants meeting any of the following conditions will be excluded from the study:

a. Severe cognitive impairment;

b. Advanced malignant tumors;

c. Life expectancy of less than 3 months;

d. Severe multi-organ failure;

e. Refusal to provide written informed consent.

3.2.3 Withdrawal Criteria and Procedures

3.2.3.1 Criteria for Withdrawal

Participants will be withdrawn from the study under any of the following circumstances:

a. Worsening of symptoms or development of clinical complications preventing continuation of treatment;

b. Treatment discontinuation due to poor efficacy or adverse reactions;

c. Participant elects to pursue alternative therapies outside the study protocol;

d. Voluntary withdrawal from the study for any reason.

3.2.3.2 Withdrawal Procedures

Withdrawal may be initiated by either the participant or the investigator. The time and reason for withdrawal will be documented.

For participants who withdraw without stating a reason, investigators will make reasonable efforts to understand the motivation while fully respecting the participant's autonomy and right to withdraw.

3.3 Interventions

Intervention Group: AIM-CHD Management System

Participants in the intervention arm will receive standard discharge instructions supplemented by digital management through the AIM-CHD (Artificial Intelligence–enhanced Management for Coronary Heart Disease) platform, delivered via a WeChat Mini Program.
The AIM-CHD system is seamlessly integrated with the hospital's electronic health record (EHR) to enable real-time data synchronization. It incorporates multiple modules, including patient follow-up questionnaires, laboratory report recognition (OCR + large language model), wearable-device monitoring, AI-assisted voice follow-up, and individualized risk stratification.

- **Training and Onboarding:**
  Participants will undergo approximately 10 minutes of in-person training conducted by research staff, including a live demonstration of key system functions and a simulated practice session to confirm operational proficiency.

- **Follow-up Mechanism:**
  Two formal follow-up assessments will be conducted at 1 month and 3 months post-discharge. The system automatically delivers questionnaires and voice reminders based on dynamic risk-stratification results.
  If suboptimal control of physiological parameters (e.g., blood pressure, heart rate, or glucose level) is detected, the system shortens the monitoring interval to every 3 days until targets are achieved.

- **Real-time Monitoring and Emergency Response:**
  When the system identifies adverse events or warning signals—such as blood pressure ≥ 160/90 mm Hg or hypoglycemia ≤ 4 mmol/L—it immediately triggers an in-app alert prompting the patient to contact the study team or initiate online emergency referral.

- **Refined Risk-Factor Management:**
  The platform integrates lifestyle and clinical indicators to provide quantitative assessments of LDL-C, blood pressure, glucose, body weight, smoking, alcohol consumption, and medication adherence.

When targets are not met, the system automatically generates tailored prompts (e.g., “LDL-C > 1.8 mmol/L—please consult your physician for possible lipid-lowering intensification”).

- **Health-Education Module:**
  Educational content—delivered through videos and infographics—is dynamically tailored to patient characteristics, covering four major domains: disease understanding, medication adherence, lifestyle modification, and cardiac rehabilitation. Content is updated quarterly.

- **Medication Reminders and Behavior Reinforcement:**
  Personalized reminders are scheduled through app notifications and AI-generated voice messages, with adjustable timing and frequency to enhance adherence.

Control Group: Usual Post-Discharge Care

Participants in the control group will receive standard oral and written discharge instructions, including guidance on medications, follow-up visits, and lifestyle recommendations, without access to the AIM-CHD system.
To ensure data consistency, participants in this group will enter outcome data at the 3-month assessment via a restricted version of the software interface, which allows data entry only—without access to risk-stratification, educational, or reminder functionalities.

3.4 Study Flow

3.4.1 Study Flowchart

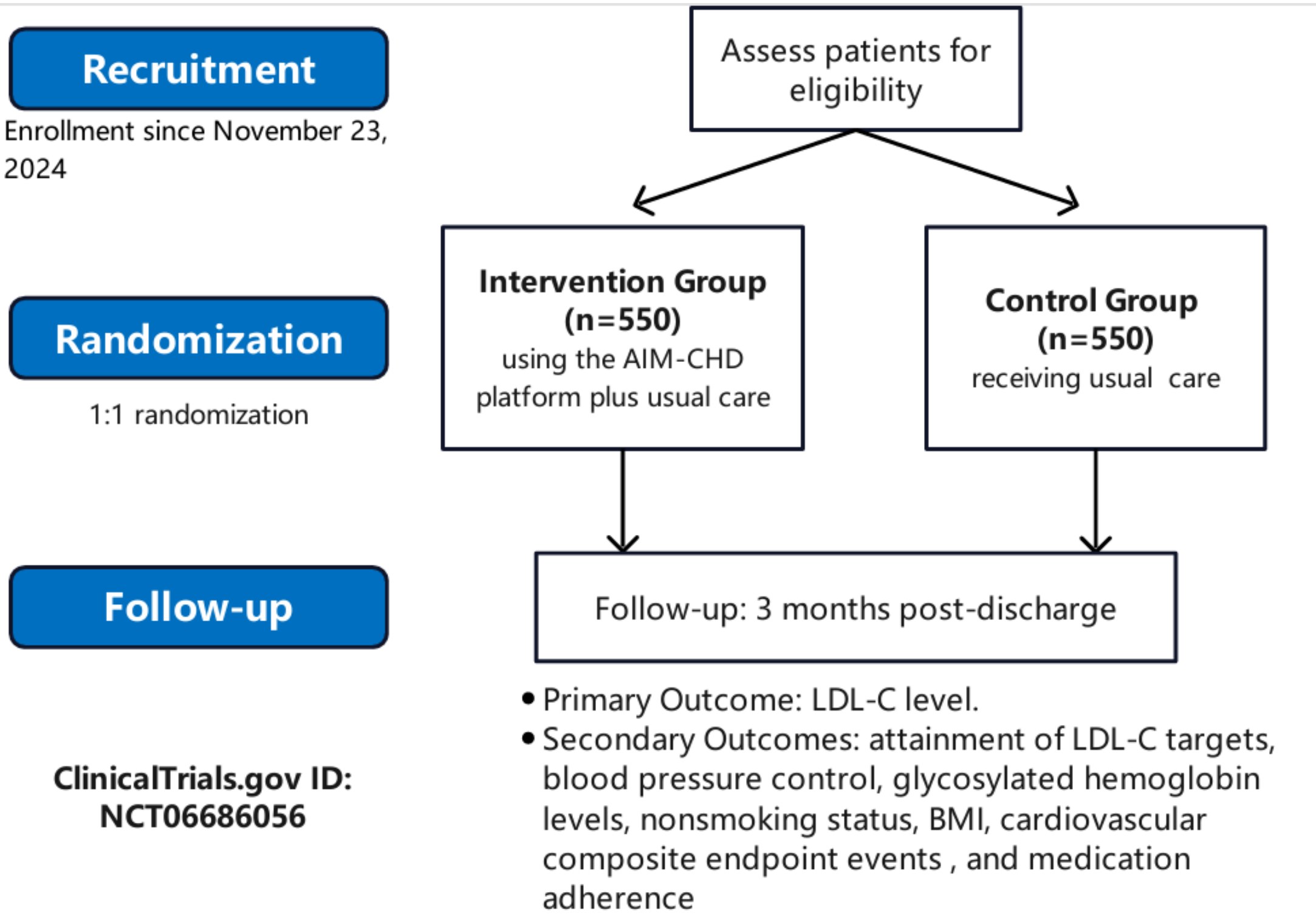

Figure 1. Study flowchart of the AIM-CHD randomized controlled trial.

3.4.2 Visit Schedule

3.4.2.1 Screening / Admission or Discharge (Visit 1)

During hospitalisation or on the day of discharge, the following procedures will be conducted:

- Investigators will explain the study objectives, procedures, and potential risks to the patient, who will then sign the written informed consent form.
- Eligibility will be assessed according to the predefined inclusion and exclusion criteria.
- Baseline laboratory data—including LDL-C, glucose, and renal function—will be automatically extracted from the hospital information system (HIS). Patients without a valid LDL-C result within the preceding 2 months will not be eligible for enrolment.
- The study coordinator will verify that the patient or a family member is capable of operating a smartphone and mobile application.

- A preliminary demonstration of the AIM-CHD system will be provided to ensure that the patient can perform basic functions such as completing questionnaires, uploading laboratory reports, and responding to voice calls.

Patients meeting all criteria and providing informed consent will proceed to randomisation.

3.4.2.2 Randomisation and Discharge Visit (Visit 2)

On the day of discharge, the following procedures will be completed:

- Confirm that discharge medications and treatment plans are stable.
- Perform central randomisation in a 1:1 ratio to assign participants to either the AIM-CHD intervention group or the usual-care control group. Randomisation will be stratified by LDL-C level at discharge (<1.8 mmol/L vs. ≥1.8 mmol/L).

For the AIM-CHD group:

- The study nurse will guide installation of the AIM-CHD Mini Program.
- A 10-minute hands-on training session will be conducted, covering the risk-stratification dashboard, questionnaire completion, laboratory report upload, and connection of wearable devices.
- A printed quick-start manual and technical support hotline number will be provided.

For the control group:

- Participants will receive standardised oral and written discharge education, including medication instructions, dietary and physical activity advice, and recommended follow-up schedules.
- The AIM-CHD system will not be activated; only a restricted data-entry interface will be retained for endpoint data collection at 3 months.

After this visit, participants will enter the active follow-up phase.

3.4.2.3 Final Evaluation Visit at 3 Months (Visit 3: Month 3 ±14 Days)

Approximately 90 ± 14 days after discharge, participants will complete the final assessment, corresponding to the primary endpoint evaluation. Procedures include:

- Completing an in-app questionnaire or AI voice follow-up to record smoking and alcohol use during the previous month and assess medication adherence.
- Measuring LDL-C and glycated haemoglobin (HbA1c) at a nearby medical facility, uploading report photos via the app. The system will automatically extract and verify data using OCR and large language model (LLM) recognition; alternatively, data may be synchronised from the hospital EHR or entered via the app questionnaire.
- Recording home-monitored blood pressure, body weight, and heart rate, obtained via hospital data synchronisation, app entry, or wearable devices.
- Documenting composite cardiovascular events within the 3-month follow-up (all-cause death, non-fatal myocardial infarction, stroke, or hospital readmission).
- Completing the System Usability Scale (SUS) questionnaire to assess user experience.
- Investigators will verify data completeness and perform telephone follow-up for missing reports or incomplete uploads.

Upon completion of this visit, participants will exit the intervention phase and proceed to data cleaning and statistical analysis.

3.4.2.4 Inter-Visit Communication and Adherence Maintenance

- The system will automatically send voice or WeChat reminders at −5, −3, 0, and +3 days relative to each scheduled follow-up.
- If no response is received, study staff will conduct a telephone reminder within +7 days.
- A dedicated technical support hotline will remain available throughout the study to assist participants with app usage or data-upload issues.

3.4.3 Randomisation Procedure

Randomisation will be conducted through a centralised, computer-generated allocation system. All eligible patients who meet the inclusion criteria and provide written informed consent will be randomised after confirmation of their discharge orders. An independent study coordinator will log into a secure web-based randomisation platform to complete group allocation.

The system will automatically retrieve each participant's most recent low-density lipoprotein cholesterol (LDL-C) result from the hospital database and assign them in a 1:1 ratio to either the AIM-CHD intervention group or the usual-care control group.

To minimise baseline imbalances, a stratified permuted-block randomisation method will be used, with discharge LDL-C level (<1.8 mmol/L vs. ≥1.8 mmol/L) as the stratification factor. The block size will be set at 4.

The randomisation sequence will be independently generated and securely encrypted by the hospital's Information Center. Investigators and outcome assessors will have no access to, or ability to modify, the randomisation sequence, thereby ensuring allocation concealment.

Group assignment will be automatically displayed to the study coordinator only after the participant has completed all discharge preparations, enabling initiation of the assigned intervention.

3.4.4 Concomitant Treatments (Medications and Devices)

All concomitant medical and device therapies will follow current national clinical practice guidelines for the secondary prevention of coronary heart disease (CHD), including:

- *Guideline for the Diagnosis and Management of Chronic Coronary Syndrome in China*;
- *Guideline for the Diagnosis and Treatment of Non–ST-Segment Elevation Acute Coronary Syndromes (2024)*; and
- *Guideline for the Diagnosis and Treatment of ST-Segment Elevation Myocardial Infarction (2019)*.

These guidelines will be used to ensure standardised and evidence-based management across all participants.

3.5 Criteria for Study Suspension or Termination

If the AIM-CHD system detects a severe abnormality—such as systolic blood pressure (SBP) > 160 mmHg, diastolic blood pressure (DBP) < 60 mmHg, heart rate (HR) > 100 beats per minute, or fasting blood glucose ≥ 14 mmol/L—an automatic in-app alert will be immediately

triggered, prompting the participant to contact the study team or seek emergency care at the nearest medical facility.

The study investigators will conduct a remote clinical assessment to confirm the patient's condition and determine whether the intervention should be temporarily suspended or permanently terminated.

All adverse events and safety signals will be graded and documented according to the Common Terminology Criteria for Adverse Events (CTCAE) version 5.0 and reported to the institutional ethics committee in accordance with regulatory requirements.

3.6 Outcome Measures

Primary Outcome

- Low-density lipoprotein cholesterol (LDL-C) level at 3 months after discharge.

Key Secondary Outcome

- Proportion of participants achieving target LDL-C, defined as LDL-C < 1.8 mmol/L at 3 months post-discharge.

Secondary Outcomes

a. Blood pressure control rate at 3 months after discharge;
b. Body mass index (BMI) at 3 months after discharge;
c. Current smoking rate at 3 months after discharge;
d. Glycated haemoglobin (HbA1c) level at 3 months after discharge;
e. Composite cardiovascular endpoint within 3 months, defined as a combination of all-cause death, non-fatal myocardial infarction, stroke, or hospital readmission;
f. Medication adherence to antiplatelet and statin therapy at 3 months after discharge, defined as self-reported adherence of ≥80% of days during the preceding month for each medication class.

3.7 Measures to Minimise Bias

To ensure methodological rigor and internal validity, the following measures will be implemented to minimise potential sources of bias:

a. Standardised data collection procedures: Comprehensive and detailed data collection protocols will be established and strictly followed throughout the study.
b. Accuracy and confidentiality of randomisation: The randomisation process will be centrally managed, computer-generated, and encrypted to ensure both precision and allocation concealment.
c. Blinding during data collection: Wherever feasible, data collection and outcome assessment will be conducted under blinded conditions to reduce observer bias.
d. Use of objective outcome measures: Laboratory test results, clinical examination records, and other verifiable data will be prioritised as objective endpoints.
e. Strict eligibility criteria: Inclusion and exclusion criteria will be rigorously applied to ensure that enrolled participants are representative of the target CHD population.
f. Participant engagement and retention: Multiple strategies—including regular reminders, flexible communication channels, and real-time technical support—will be used to maximise cooperation, minimise non-response, and reduce loss to follow-up.
g. Control of confounding bias: Matching, stratified analysis, and multivariable statistical modelling will be employed to adjust for potential confounders.

4. Statistical Considerations

4.1 Sample Size Estimation

This study adopts a prospective, single-centre, randomised controlled design and is structured as a superiority trial. The sample size was determined based on the primary outcome—mean low-density lipoprotein cholesterol (LDL-C) level at 3 months—and on assumptions regarding the expected treatment effect, according to standard statistical principles.

Recent studies of Chinese coronary heart disease (CHD) populations receiving secondary prevention therapy have reported mean LDL-C levels ranging from 2.1 to 2.7 mmol/L, with standard deviations (SDs) between 0.80 and 0.95 mmol/L (Cardiovasc Diabetol. 2024;23:190; 2023;22:161; 2022;21:168; 2024;23:79).

For the present trial, it was assumed that the mean LDL-C level at 3 months in the intervention group would be 0.20 mmol/L (approximately 10%) lower than that in the control group, with an SD of 0.95 mmol/L. Based on these assumptions, using PASS software

(version 15.0) for a superiority test with a one-sided α of 0.025 and 90% power ($1-\beta = 0.90$), the required sample size was calculated to be 950 participants.

Allowing for an anticipated 10% attrition rate, the final target sample size for this study is 1,100 participants.

4.2 Statistical Analysis Plan

The statistical analysis will adhere to the intention-to-treat (ITT) principle, following a prespecified plan approved prior to unblinding.

Analysis Populations

Two analysis sets will be defined:

- Intention-to-treat (ITT) population: all randomised participants analysed as allocated, regardless of adherence.
- Per-protocol (PP) population: includes intervention participants who completed at least one lipid test and follow-up questionnaire, and all control participants who completed outcome assessments.
- Safety population: includes all participants who received at least one intervention exposure or follow-up assessment, used for safety and adverse event analyses.

Descriptive and Baseline Analyses

Continuous variables will be presented as mean (SD) or median (IQR), and categorical variables as n (%). Baseline group differences will be assessed using the t test or Wilcoxon rank-sum test for continuous data, and the χ^2 test or Fisher's exact test for categorical data.

Primary and Secondary Outcomes

- The primary outcome, LDL-C level at 3 months, will be analysed using analysis of covariance (ANCOVA) adjusted for baseline LDL-C. Results will be expressed as adjusted mean differences (AMDs) with 95% confidence intervals (CIs).

- Binary secondary outcomes (e.g., LDL-C target attainment, blood pressure control, medication adherence) will be analysed using modified Poisson regression with robust variance, reported as risk ratios (RRs) and 95% CIs.
- The composite cardiovascular endpoint (all-cause death, non-fatal myocardial infarction, stroke, or readmission) will be evaluated using Kaplan–Meier survival analysis and compared between groups via Cox proportional-hazards models, adjusting for baseline risk strata and reported as hazard ratios (HRs) with 95% CIs.
- Continuous secondary outcomes (e.g., BMI, HbA1c, blood pressure) will be analysed using ANCOVA models adjusted for baseline values.

Handling of Missing Data

Missing data will be addressed using multiple imputation ($m = 20$) under the missing-at-random assumption.
Sensitivity analyses will include complete-case and per-protocol analyses.
To assess robustness against violations of the missing-at-random assumption, additional analyses will include Δ-tipping-point analysis, inverse probability of censoring weighting (IPCW), and best–worst case scenarios.

Subgroup and Sensitivity Analyses

Predefined subgroup analyses will examine potential effect modification by sex, age, education, ethnicity, smoking status, baseline risk stratification, and baseline LDL-C target attainment. Interactions between treatment and subgroup factors will be formally tested, and p-values for interaction will be reported.

Software and Oversight

All statistical analyses will be performed using R software (version 4.4.1).
An independent Data Monitoring Committee (DMC) will provide safety oversight throughout the trial.

5. Confidentiality

Strict measures will be implemented to protect the privacy and personal information of all study participants.

1. Comprehensive data confidentiality and information security protocols have been established in accordance with institutional and regulatory requirements. All data will be stored on secure servers with restricted access, and personal identifiers will be replaced by unique coded IDs.

2. Study results may be published or presented in academic journals or scientific meetings. However, no participant's name, identification number, or any information that could directly or indirectly identify an individual will be disclosed.

All handling of participant data will comply with applicable privacy protection regulations, including institutional policies, the *Personal Information Protection Law of the People's Republic of China*, and relevant international data protection standards.

6. Quality Assurance

6.1 Case Report Form (CRF) and Tool Development

The CRFs and standard operating procedures (SOPs) for medical record extraction were developed to address the study's primary research questions. An extensive literature review and focused expert discussions ensured conceptual and terminological accuracy during data abstraction. Staff responsible for completing CRFs received structured training to ensure consistent and accurate completion of CRFs and patient-reported outcome measures. Investigators will conduct spot checks and competency assessments.

6.2 Patient Eligibility

Strictly defined inclusion criteria will be applied. Discharge diagnoses used after data extraction will be verified against unified standards to ensure consistency.

6.3 Data Entry

The electronic data capture (EDC) system transmits data from the client to the server via the internet. Investigators will enter source data directly into the EDC without paper CRFs. Entered data must reflect the authenticity and completeness of the source records.

6.4 Data Cleaning

All entered data will undergo checks for duplicate records and outliers. Potential duplicates will be adjudicated using a composite of variables (e.g., medical record ID, discharge date/time). Suspected errors will be documented in quality-control reports and issued as data queries to data monitors. When data updates or corrections are required, monitors will re-review the corresponding CRFs and/or source documents.

6.5 Data Analysis

Following data cleaning, the database will be locked, and all analyses will be performed on the final locked dataset. In accordance with the analysis plan, two independent programmers will develop analysis code separately; a senior analyst will review outputs and reconcile any discrepancies between programs.

7. Ethical Principles

7.1 Ethical Approval

1. The study will be initiated only after obtaining approval from the institutional ethics committee.
2. All study procedures will strictly adhere to the approved protocol and standard operating procedures.
3. Any amendments to the protocol or informed consent form must be reviewed and approved by the ethics committee before implementation.

7.2 Informed Consent

Before enrolment, each participant—or their legally authorised representative—will be provided with a written informed consent form that has been reviewed and approved by the ethics committee.

The form will contain comprehensive and comprehensible information regarding the study's purpose, procedures, potential risks, and expected benefits. Participants or their representatives will be given sufficient time to consider participation.

Written informed consent must be obtained prior to any study-related procedures.
If new safety information arises that may materially alter the risk–benefit assessment, participants (or their guardians) will be promptly informed and re-consented using the revised document.

7.3 Risk–Benefit Assessment

This study aims to evaluate the application value of a generative artificial intelligence (AI)–based system for innovative long-term management of coronary heart disease (CHD), introducing new strategies for post-discharge care. The novel management approach is expected to improve clinical outcomes and quality of life for CHD patients.
The intervention, developed in accordance with established clinical guidelines, represents a digital enhancement of conventional post-discharge management and carries minimal additional risk.
Throughout the study and follow-up, investigators will actively monitor for adverse events (AEs). Any AE will be assessed by the investigators, and management strategies will be adjusted if necessary. All AEs will be documented and reported as required.

7.4 Conflict of Interest

No conflicts of interest are declared by the investigators.

7.5 Participant Protection Measures

Only participants who meet all inclusion criteria and no exclusion criteria will be invited to join the study. Eligible patients will be approached before discharge, and informed consent will be sought after thorough explanation of the study details.
All collected data will exclude personally identifiable information such as names or contact details. Study documents will be securely stored in locked cabinets within the project office. All study personnel—including the principal investigator, research staff, and authorised monitors from regulatory or academic institutions—must receive data security training and obtain written authorisation before accessing source documents or databases.
No participant information will be shared with employers or insurance agencies. Strict security measures will be implemented to prevent unauthorised access to participant data, and no personally identifiable information will appear in any publications or reports.

8. Project Risk Assessment and Contingency Plan

This project primarily focuses on risk-factor management, therapeutic pathways, evaluation methods, and strategic optimisation for coronary heart disease. All study components are based on proprietary intellectual property, and therefore no market or policy risks are anticipated.

Potential risks mainly include technical, management, and pandemic-related risks.

- Technical risks:
  These may include adverse events unrelated to the intervention itself, overly strict or lenient inclusion/exclusion criteria, inappropriate selection or timing of study endpoints, insufficient sample size, or improper analytical methods—each of which could compromise trial validity.

- Management risks:
  Possible challenges include ethical review delays, inadequate personnel training, or protocol deviations.

- Pandemic-related risks:
  COVID-19–related restrictions may affect access to medical care or participant follow-up.

To mitigate these risks, the following measures will be implemented:

1. Technical reliability: The study's core technical framework will be rigorously evaluated for reliability, feasibility, and applicability to ensure optimal implementation. If investigators identify any unexpected or unacceptable risks to participants, trial suspension or termination will be considered.

2. Participant recruitment and monitoring: Consecutive enrolment of eligible participants will be prioritised to minimise selection bias. Recruitment progress and follow-up completion will be regularly monitored to ensure data integrity and completeness.

3. Protocol compliance: All study procedures will strictly adhere to the approved protocol. No deviations or substantial modifications will be made without prior

approval from the investigators, the ethics committee, and, when applicable, the National Medical Products Administration (NMPA).

9. Protocol Deviations and Amendments

Investigators will submit periodic reports to the ethics committee detailing any protocol deviations. Immediate reporting will be required in the case of major or serious violations. All deviations must be documented in writing, including a full description of the event, evaluation of its impact on participant safety and rights, and determination of whether the participant should continue or be withdrawn from the study. The potential influence of each deviation on subsequent data collection and analysis will also be assessed.

A Protocol Deviation Oversight Committee will be established to review deviation logs regularly, identify systematic trends or recurrent errors, and ensure timely corrective actions. If major deviations necessitate protocol revision, the study will be temporarily suspended until the revised protocol has been approved and all study personnel have completed retraining.

10. Study Organisation and Responsibilities

The principal investigator will conduct the study in accordance with the approved research plan and oversee all operational activities.
The Information Center, Department of Medical Statistics, and other collaborating units will independently perform their respective duties—such as data collection, management, monitoring, and analysis—to ensure the scientific integrity and overall quality of the research data.

11. References

1. National Center for Cardiovascular Diseases, China. *China Cardiovascular Health and Disease Report 2023 (Summary)*. *Chinese Circulation Journal*. 2024;39(7):625–660. (in Chinese)

2. Byrne RA, Rossello X, Coughlan JJ, et al. 2023 ESC Guidelines for the management of acute coronary syndromes. *Eur Heart J*. 2023;44(38):3720–3826.

3. Chinese Society of Cardiology; Editorial Board of *Chinese Journal of Cardiology*. *Guideline for the Diagnosis and Management of Chronic Coronary Syndrome in China*. *Chin J Cardiol*. 2024;52(6):589–614. (in Chinese)

4. Chinese Society of Cardiology; Editorial Board of *Chinese Journal of Cardiology*. *Guideline for the Diagnosis and Treatment of Non–ST-Segment Elevation Acute Coronary Syndromes (2024)*. *Chin J Cardiol*. 2024;52(6):615–646. (in Chinese)

5. Chinese Society of Cardiology; Editorial Board of *Chinese Journal of Cardiology*. *Guideline for the Diagnosis and Treatment of ST-Segment Elevation Myocardial Infarction (2019)*. *Chin J Cardiol*. 2019;47(10):766–783. (in Chinese)

6. Yusuf S, Islam S, Chow CK, et al. Use of secondary prevention drugs for cardiovascular disease in the community in high-, middle-, and low-income countries (the PURE Study): a prospective epidemiological survey. *Lancet*. 2011;378(9798):1231–1243.

7. Li HJ, Zhao D, Liu J, et al. Current use of statins for secondary prevention in Chinese patients with very high-risk coronary heart disease. *Chin J Cardiol*. 2010;38(11):5. (in Chinese)

8. Laxminarayan S, Istepanian RSH. UNWIRED E-MED: the next generation of wireless and internet telemedicine systems. *IEEE Trans Inf Technol Biomed*. 2000;4(3):189–193.

9. Godinho MA, Jonnagaddala J, Gudi N, et al. mHealth for Integrated People-Centred Health Services in the Western Pacific: a systematic review. *Int J Med Inform*. 2020;142:104259.

10. Jiang X, Ming WK, You JH. The cost-effectiveness of digital health interventions in the management of cardiovascular diseases: systematic review. *J Med Internet Res*. 2019;21(6):e13166.

11. Sarwar CMS, Vaduganathan M, Anker SD, et al. Mobile health applications in cardiovascular research. *Int J Cardiol*. 2018;269:265–271.

12. Al-Arkee S, Mason J, Lane DA, et al. Mobile apps to improve medication adherence in cardiovascular disease: systematic review and meta-analysis. *J Med Internet Res*. 2021;23(5):e24190.

13. Bernal-Jiménez MA, Calle G, Gutiérrez Barrios A, et al. Effectiveness of an interactive mHealth app (EVITE) in improving lifestyle after a coronary event: randomized controlled trial. *JMIR Mhealth Uhealth*. 2024;12:e48756.

14. Chow CK, Redfern J, Hillis GS, et al. Effect of lifestyle-focused text messaging on risk factor modification in patients with coronary heart disease: a randomized clinical trial. *JAMA*. 2015;314(12):1255–1263.

15. Golbus JR, Shi J, Gupta K, et al. Text messages to promote physical activity in patients with cardiovascular disease: a micro-randomized trial of a just-in-time adaptive intervention. *Circ Cardiovasc Qual Outcomes*. 2024;17(7):e010731.

16. Fang R, Li X. Electronic messaging support service programs improve adherence to lipid-lowering therapy among outpatients with coronary artery disease: an exploratory randomized controlled study. *J Clin Nurs*. 2016;25(5–6):664–671.

17. Ni Z, Wu B, Yang Q, et al. An mHealth intervention to improve medication adherence and health outcomes among patients with coronary heart disease: randomized controlled trial. *J Med Internet Res*. 2022;24(3):e27202.

18. Thom SJM, Sivakumar B, Ayodele T, et al. Impact of mHealth interventions on supporting dietary adherence in cardiovascular disease: a systematic review. *J Nutr Educ Behav*. 2023;55(6):419–436.

19. Chow CK, Thiagalingam A, Santo K, et al. TEXT messages to improve MEDication adherence and Secondary prevention (TEXTMEDS) after acute coronary syndrome: a randomized clinical trial protocol. *BMJ Open*. 2018;8(1):e019463.

20. Chow CK, Klimis H, Thiagalingam A, et al. Text messages to improve medication adherence and secondary prevention after acute coronary syndrome: the TEXTMEDS randomized clinical trial. *Circulation*. 2022;145(19):1443–1455.

21. Yu C, Liu C, Du J, et al. Smartphone-based application to improve medication adherence in patients after surgical coronary revascularization. *Am Heart J*. 2020;228:17–26.

22. Fuwai Hospital, Chinese Academy of Medical Sciences. *Coronary Heart Disease Post-PCI Health Enhancement Program System V1.6.8* [software]. Registration No. 2023SR1362386. National Copyright Administration; 2023. (in Chinese)

Section/topic	No	CONSORT 2025 checklist item description	Reported on page no.
**Title and abstract**			
Title and structured abstract	1a	Identification as a randomised trial	1
	1b	Structured summary of the trial design, methods, results, and conclusions	1-3
**Open science**			
Trial registration	2	Name of trial registry, identifying number (with URL) and date of registration	3
Protocol and statistical analysis plan	3	Where the trial protocol and statistical analysis plan can be accessed	4-5, Additional file 1
Data sharing	4	Where and how the individual de-identified participant data (including data dictionary), statistical code and any other materials can be accessed	17
Funding and conflicts of interest	5a	Sources of funding and other support (eg, supply of drugs), and role of funders in the design, conduct, analysis and reporting of the trial	17
	5b	Financial and other conflicts of interest of the manuscript authors	18
**Introduction**			
Background and rationale	6	Scientific background and rationale	4
Objectives	7	Specific objectives related to benefits and harms	4
**Methods**			
Patient and public involvement	8	Details of patient or public involvement in the design, conduct and reporting of the trial	Additional file 1 (protocol)
Trial design	9	Description of trial design including type of trial (eg, parallel group, crossover), allocation ratio, and framework (eg, superiority, equivalence, non-inferiority, exploratory)	4
Changes to trial protocol	10	Important changes to the trial after it commenced including any outcomes or analyses that were not prespecified, with reason	NA
Trial setting	11	Settings (eg, community, hospital) and locations (eg, countries, sites) where the trial was conducted	4, 9
Eligibility criteria	12a	Eligibility criteria for participants	5
	12b	If applicable, eligibility criteria for sites and for individuals delivering the interventions (eg, surgeons, physiotherapists)	5
Intervention and comparator	13	Intervention and comparator with sufficient details to allow replication. If relevant, where additional materials describing the intervention and comparator (eg, intervention manual) can be accessed	5-7, Additional file 1
Outcomes	14	Prespecified primary and secondary outcomes, including the specific measurement variable (eg, systolic blood pressure), analysis metric (eg, change from baseline, final value, time to event), method of aggregation (eg, median, proportion), and time point for each outcome	7
Harms	15	How harms were defined and assessed (eg, systematically, non-systematically)	7, Additional file 1
Sample size	16a	How sample size was determined, including all assumptions supporting the sample size calculation	8
	16b	Explanation of any interim analyses and stopping guidelines	NA
Randomisation:			

Sequence generation	17a	Who generated the random allocation sequence and the method used	5
	17b	Type of randomisation and details of any restriction (eg, stratification, blocking and block size)	5
Allocation concealment mechanism	18	Mechanism used to implement the random allocation sequence (eg, central computer/telephone; sequentially numbered, opaque, sealed containers), describing any steps to conceal the sequence until interventions were assigned	5
Implementation	19	Whether the personnel who enrolled and those who assigned participants to the interventions had access to the random allocation sequence	5, Additional file 1 (protocol)
Blinding	20a	Who was blinded after assignment to interventions (eg, participants, care providers, outcome assessors, data analysts)	5
	20b	If blinded, how blinding was achieved and description of the similarity of interventions	NA
Statistical methods	21a	Statistical methods used to compare groups for primary and secondary outcomes, including harms	8
	21b	Definition of who is included in each analysis (eg, all randomised participants), and in which group	8
	21c	How missing data were handled in the analysis	8
	21d	Methods for any additional analyses (eg, subgroup and sensitivity analyses), distinguishing prespecified from post hoc	8-9
**Results**			
Participant flow, including flow diagram	22a	For each group, the numbers of participants who were randomly assigned, received intended intervention, and were analysed for the primary outcome	9, Fig. 2
	22b	For each group, losses and exclusions after randomisation, together with reasons	9, Fig. 2
Recruitment	23a	Dates defining the periods of recruitment and follow-up for outcomes of benefits and harms	9
	23b	If relevant, why the trial ended or was stopped	NA
Intervention and comparator delivery	24a	Intervention and comparator as they were actually administered (eg, where appropriate, who delivered the intervention/comparator, how participants adhered, whether they were delivered as intended (fidelity))	10-11
	24b	Concomitant care received during the trial for each group	NA
Baseline data	25	A table showing baseline demographic and clinical characteristics for each group	9, Table 1
Numbers analysed, outcomes and estimation	26	For each primary and secondary outcome, by group:<br>● the number of participants included in the analysis<br>● the number of participants with available data at the outcome time point<br>● result for each group, and the estimated effect size and its precision (such as 95% confidence interval)<br>● for binary outcomes, presentation of both absolute and relative effect size	Tables 2-3
Harms	27	All harms or unintended events in each group	11, Additional file 1
Ancillary analyses	28	Any other analyses performed, including subgroup and sensitivity analyses, distinguishing pre-specified from post hoc	10-11, Additional file 1
**Discussion**			
Interpretation	29	Interpretation consistent with results, balancing benefits and harms, and considering other relevant evidence	11-14
Limitations	30	Trial limitations, addressing sources of potential bias, imprecision, generalisability, and, if relevant, multiplicity of analyses	14

Citation: Hopewell S, Chan AW, Collins GS, Hróbjartsson A, Moher D, Schulz KF, et al. CONSORT 2025 Statement: updated guideline for reporting randomised trials. BMJ. 2025; 388:e081123. https://dx.doi.org/10.1136/bmj-2024-081123

© 2025 Hopewell et al. This is an Open Access article distributed under the terms of the Creative Commons Attribution License (https://creativecommons.org/licenses/by/4.0/), which permits unrestricted use, distribution, and reproduction in any medium, provided the original work is properly cited.

*We strongly recommend reading this statement in conjunction with the CONSORT 2025 Explanation and Elaboration and/or the CONSORT 2025 Expanded Checklist for important clarifications on all the items. We also recommend reading relevant CONSORT extensions. See www.consort-spirit.org.
